# Search for dark matter, extra dimensions, and unparticles in monojet events in proton–proton collisions at $$\sqrt{s} = 8$$$$\,{\mathrm{TeV}}\,$$

**DOI:** 10.1140/epjc/s10052-015-3451-4

**Published:** 2015-05-29

**Authors:** V. Khachatryan, A. M. Sirunyan, A. Tumasyan, W. Adam, T. Bergauer, M. Dragicevic, J. Erö, C. Fabjan, M. Friedl, R. Frühwirth, V. M. Ghete, C. Hartl, N. Hörmann, J. Hrubec, M. Jeitler, W. Kiesenhofer, V. Knünz, M. Krammer, I. Krätschmer, D. Liko, I. Mikulec, D. Rabady, B. Rahbaran, H. Rohringer, R. Schöfbeck, J. Strauss, A. Taurok, W. Treberer-Treberspurg, W. Waltenberger, C.-E. Wulz, V. Mossolov, N. Shumeiko, J. SuarezGonzalez, S. Alderweireldt, M. Bansal, S. Bansal, T. Cornelis, E. A. De Wolf, X. Janssen, A. Knutsson, S. Luyckx, S. Ochesanu, B. Roland, R. Rougny, M. Van De Klundert, H. Van Haevermaet, P. Van Mechelen, N. Van Remortel, A. Van Spilbeeck, F. Blekman, S. Blyweert, J. D’Hondt, N. Daci, N. Heracleous, A. Kalogeropoulos, J. Keaveney, T.J. Kim, S. Lowette, M. Maes, A. Olbrechts, Q. Python, D. Strom, S. Tavernier, W. Van Doninck, P. Van Mulders, G. P. Van Onsem, I. Villella, C. Caillol, B. Clerbaux, G. De Lentdecker, D. Dobur, L. Favart, A. P. R. Gay, A. Grebenyuk, A. Léonard, A. Mohammadi, L. Perniè, T. Reis, T. Seva, L. Thomas, C. Vander Velde, P. Vanlaer, J. Wang, V. Adler, K. Beernaert, L. Benucci, A. Cimmino, S. Costantini, S. Crucy, S. Dildick, A. Fagot, G. Garcia, B. Klein, J. Mccartin, A. A. Ocampo Rios, D. Ryckbosch, S. Salva Diblen, M. Sigamani, N. Strobbe, F. Thyssen, M. Tytgat, E. Yazgan, N. Zaganidis, S. Basegmez, C. Beluffi, G. Bruno, R. Castello, A. Caudron, L. Ceard, G. G. Da Silveira, C. Delaere, T. du Pree, D. Favart, L. Forthomme, A. Giammanco, J. Hollar, P. Jez, M. Komm, V. Lemaitre, J. Liao, C. Nuttens, D. Pagano, L. Perrini, A. Pin, K. Piotrzkowski, A. Popov, L. Quertenmont, M. Selvaggi, M. Vidal Marono, J. M. Vizan Garcia, N. Beliy, T. Caebergs, E. Daubie, G. H. Hammad, W. L. Aldá Júnior, G. A. Alves, M. CorreaMartins Junior, T. Dos Reis Martins, M. E. Pol, W. Carvalho, J. Chinellato, A. Custódio, E. M. Da Costa, D. De JesusDamiao, C. De OliveiraMartins, S. Fonseca De Souza, H. Malbouisson, M. Malek, D. MatosFigueiredo, L. Mundim, H. Nogima, W. L. Prado DaSilva, J. Santaolalla, A. Santoro, A. Sznajder, E. J. Tonelli Manganote, A. Vilela Pereira, C. A. Bernardes, S. Dogra, F.A. Dias, T. R. FernandezPerez Tomei, E. M. Gregores, P. G. Mercadante, S. F. Novaes, Sandra S. Padula, A. Aleksandrov, V. Genchev, P. Iaydjiev, A. Marinov, S. Piperov, M. Rodozov, G. Sultanov, M. Vutova, A. Dimitrov, I. Glushkov, R. Hadjiiska, V. Kozhuharov, L. Litov, B. Pavlov, P. Petkov, J. G. Bian, G. M. Chen, H. S. Chen, M. Chen, R. Du, C. H. Jiang, D. Liang, S. Liang, R. Plestina, J. Tao, X. Wang, Z. Wang, C. Asawatangtrakuldee, Y. Ban, Y. Guo, Q. Li, W. Li, S. Liu, Y. Mao, S. J. Qian, D. Wang, L. Zhang, W. Zou, C. Avila, L. F. Sierra Chaparro, C. Florez, J. P. Gomez, B. Gomez Moreno, J. C. Sanabria, N. Godinovic, D. Lelas, D. Polic, I. Puljak, Z. Antunovic, M. Kovac, V. Brigljevic, K. Kadija, J. Luetic, D. Mekterovic, L. Sudic, A. Attikis, G. Mavromanolakis, J. Mousa, C. Nicolaou, F. Ptochos, P. A. Razis, M. Bodlak, M. Finger, M. Finger, Y. Assran, S. Elgammal, M. A. Mahmoud, A. Radi, B. Calpas, M. Kadastik, M. Murumaa, M. Raidal, A. Tiko, P. Eerola, G. Fedi, M. Voutilainen, J. Härkönen, V. Karimäki, R. Kinnunen, M. J. Kortelainen, T. Lampén, K. Lassila-Perini, S. Lehti, T. Lindén, P. Luukka, T. Mäenpää, T. Peltola, E. Tuominen, J. Tuominiemi, E. Tuovinen, L. Wendland, T. Tuuva, M. Besancon, F. Couderc, M. Dejardin, D. Denegri, B. Fabbro, J. L. Faure, C. Favaro, F. Ferri, S. Ganjour, A. Givernaud, P. Gras, G. Hamel de Monchenault, P. Jarry, E. Locci, J. Malcles, A. Nayak, J. Rander, A. Rosowsky, M. Titov, S. Baffioni, F. Beaudette, P. Busson, C. Charlot, T. Dahms, M. Dalchenko, L. Dobrzynski, N. Filipovic, A. Florent, R. Granier de Cassagnac, L. Mastrolorenzo, P. Miné, C. Mironov, I. N. Naranjo, M. Nguyen, C. Ochando, S. Regnard, R. Salerno, J. B. Sauvan, Y. Sirois, C. Veelken, Y. Yilmaz, A. Zabi, J.-L. Agram, J. Andrea, A. Aubin, D. Bloch, J.-M. Brom, E. C. Chabert, C. Collard, E. Conte, J.-C. Fontaine, D. Gelé, U. Goerlach, C. Goetzmann, A.-C. Le Bihan, P. Van Hove, S. Gadrat, S. Beauceron, N. Beaupere, G. Boudoul, S. Brochet, C. A. Carrillo Montoya, J. Chasserat, R. Chierici, D. Contardo, P. Depasse, H. ElMamouni, J. Fan, J. Fay, S. Gascon, M. Gouzevitch, B. Ille, T. Kurca, M. Lethuillier, L. Mirabito, S. Perries, J. D. Ruiz Alvarez, D. Sabes, L. Sgandurra, V. Sordini, M. Vander Donckt, P. Verdier, S. Viret, H. Xiao, L. Rurua, C. Autermann, S. Beranek, M. Bontenackels, M. Edelhoff, L. Feld, O. Hindrichs, K. Klein, A. Ostapchuk, A. Perieanu, F. Raupach, J. Sammet, S. Schael, D. Sprenger, H. Weber, B. Wittmer, V. Zhukov, M. Ata, J. Caudron, E. Dietz-Laursonn, D. Duchardt, M. Erdmann, R. Fischer, A. Güth, T. Hebbeker, C. Heidemann, K. Hoepfner, D. Klingebiel, S. Knutzen, P. Kreuzer, M. Merschmeyer, A. Meyer, M. Olschewski, K. Padeken, P. Papacz, H. Reithler, S. A. Schmitz, L. Sonnenschein, D. Teyssier, S. Thüer, M. Weber, V. Cherepanov, Y. Erdogan, G. Flügge, H. Geenen, M. Geisler, W. Haj Ahmad, F. Hoehle, B. Kargoll, T. Kress, Y. Kuessel, J. Lingemann, A. Nowack, I. M. Nugent, L. Perchalla, O. Pooth, A. Stahl, I. Asin, N. Bartosik, J. Behr, W. Behrenhoff, U. Behrens, A. J. Bell, M. Bergholz, A. Bethani, K. Borras, A. Burgmeier, A. Cakir, L. Calligaris, A. Campbell, S. Choudhury, F. Costanza, C. Diez Pardos, S. Dooling, T. Dorland, G. Eckerlin, D. Eckstein, T. Eichhorn, G. Flucke, J. Garay Garcia, A. Geiser, P. Gunnellini, J. Hauk, G. Hellwig, M. Hempel, D. Horton, H. Jung, M. Kasemann, P. Katsas, J. Kieseler, C. Kleinwort, D. Krücker, W. Lange, J. Leonard, K. Lipka, A. Lobanov, W. Lohmann, B. Lutz, R. Mankel, I. Marfin, I.-A. Melzer-Pellmann, A. B. Meyer, J. Mnich, A. Mussgiller, S. Naumann-Emme, O. Novgorodova, F. Nowak, E. Ntomari, H. Perrey, D. Pitzl, R. Placakyte, A. Raspereza, P. M. Ribeiro Cipriano, E. Ron, M. Ö. Sahin, J. Salfeld-Nebgen, P. Saxena, R. Schmidt, T. Schoerner-Sadenius, M. Schröder, S. Spannagel, A. D. R. VargasTrevino, R. Walsh, C. Wissing, M. Aldaya Martin, V. Blobel, M. CentisVignali, J. Erfle, E. Garutti, K. Goebel, M. Görner, M. Gosselink, J. Haller, R. S. Höing, H. Kirschenmann, R. Klanner, R. Kogler, J. Lange, T. Lapsien, T. Lenz, I. Marchesini, J. Ott, T. Peiffer, N. Pietsch, D. Rathjens, C. Sander, H. Schettler, P. Schleper, E. Schlieckau, A. Schmidt, M. Seidel, J. Sibille, V. Sola, H. Stadie, G. Steinbrück, D. Troendle, E. Usai, L. Vanelderen, C. Barth, C. Baus, J. Berger, C. Böser, E. Butz, T. Chwalek, W. De Boer, A. Descroix, A. Dierlamm, M. Feindt, F. Frensch, F. Hartmann, T. Hauth, U. Husemann, I. Katkov, A. Kornmayer, E. Kuznetsova, P. LobellePardo, M. U. Mozer, Th. Müller, A. Nürnberg, G. Quast, K. Rabbertz, F. Ratnikov, S. Röcker, H. J. Simonis, F. M. Stober, R. Ulrich, J. Wagner-Kuhr, S. Wayand, T. Weiler, R. Wolf, G. Anagnostou, G. Daskalakis, T. Geralis, V. A. Giakoumopoulou, A. Kyriakis, D. Loukas, A. Markou, C. Markou, A. Psallidas, I. Topsis-Giotis, A. Panagiotou, N. Saoulidou, E. Stiliaris, X. Aslanoglou, I. Evangelou, G. Flouris, C. Foudas, P. Kokkas, N. Manthos, I. Papadopoulos, E. Paradas, G. Bencze, C. Hajdu, P. Hidas, D. Horvath, F. Sikler, V. Veszpremi, G. Vesztergombi, A. J. Zsigmond, N. Beni, S. Czellar, J. Karancsi, J. Molnar, J. Palinkas, Z. Szillasi, P. Raics, Z. L. Trocsanyi, B. Ujvari, S. K. Swain, S. B. Beri, V. Bhatnagar, N. Dhingra, R. Gupta, A. K. Kalsi, M. Kaur, M. Mittal, N. Nishu, J. B. Singh, Ashok Kumar, Arun Kumar, S. Ahuja, A. Bhardwaj, B. C. Choudhary, A. Kumar, S. Malhotra, M. Naimuddin, K. Ranjan, V. Sharma, S. Banerjee, S. Bhattacharya, K. Chatterjee, S. Dutta, B. Gomber, Sa. Jain, Sh. Jain, R. Khurana, A. Modak, S. Mukherjee, D. Roy, S. Sarkar, M. Sharan, A. Abdulsalam, D. Dutta, S. Kailas, V. Kumar, A. K. Mohanty, L. M. Pant, P. Shukla, A. Topkar, T. Aziz, S. Banerjee, R. M. Chatterjee, R. K. Dewanjee, S. Dugad, S. Ganguly, S. Ghosh, M. Guchait, A. Gurtu, G. Kole, S. Kumar, M. Maity, G. Majumder, K. Mazumdar, G. B. Mohanty, B. Parida, K. Sudhakar, N. Wickramage, H. Bakhshiansohi, H. Behnamian, S. M. Etesami, A. Fahim, R. Goldouzian, A. Jafari, M. Khakzad, M. Mohammadi Najafabadi, M. Naseri, S. Paktinat Mehdiabadi, B. Safarzadeh, M. Zeinali, M. Felcini, M. Grunewald, M. Abbrescia, L. Barbone, C. Calabria, S. S. Chhibra, A. Colaleo, D. Creanza, N. De Filippis, M. DePalma, L. Fiore, G. Iaselli, G. Maggi, M. Maggi, S. My, S. Nuzzo, A. Pompili, G. Pugliese, R. Radogna, G. Selvaggi, L. Silvestris, G. Singh, R. Venditti, P. Verwilligen, G. Zito, G. Abbiendi, A. C. Benvenuti, D. Bonacorsi, S. Braibant-Giacomelli, L. Brigliadori, R. Campanini, P. Capiluppi, A. Castro, F. R. Cavallo, G. Codispoti, M. Cuffiani, G. M. Dallavalle, F. Fabbri, A. Fanfani, D. Fasanella, P. Giacomelli, C. Grandi, L. Guiducci, S. Marcellini, G. Masetti, A. Montanari, F. L. Navarria, A. Perrotta, A. M. Rossi, F. Primavera, T. Rovelli, G. P. Siroli, N. Tosi, R. Travaglini, S. Albergo, G. Cappello, M. Chiorboli, S. Costa, F. Giordano, R. Potenza, A. Tricomi, C. Tuve, G. Barbagli, V. Ciulli, C. Civinini, R. D’Alessandro, E. Focardi, E. Gallo, S. Gonzi, V. Gori, P. Lenzi, M. Meschini, S. Paoletti, G. Sguazzoni, A. Tropiano, L. Benussi, S. Bianco, F. Fabbri, D. Piccolo, F. Ferro, M. LoVetere, E. Robutti, S. Tosi, M. E. Dinardo, S. Fiorendi, S. Gennai, R. Gerosa, A. Ghezzi, P. Govoni, M. T. Lucchini, S. Malvezzi, R. A. Manzoni, A. Martelli, B. Marzocchi, D. Menasce, L. Moroni, M. Paganoni, D. Pedrini, S. Ragazzi, N. Redaelli, T. Tabarelli de Fatis, S. Buontempo, N. Cavallo, S. Di Guida, F. Fabozzi, A. O. M. Iorio, L. Lista, S. Meola, M. Merola, P. Paolucci, P. Azzi, N. Bacchetta, D. Bisello, A. Branca, P. Checchia, M. Dall’Osso, T. Dorigo, U. Dosselli, M. Galanti, F. Gasparini, U. Gasparini, A. Gozzelino, K. Kanishchev, S. Lacaprara, M. Margoni, A. T. Meneguzzo, M. Passaseo, J. Pazzini, M. Pegoraro, N. Pozzobon, P. Ronchese, F. Simonetto, E. Torassa, M. Tosi, P. Zotto, A. Zucchetta, G. Zumerle, M. Gabusi, S. P. Ratti, C. Riccardi, P. Salvini, P. Vitulo, M. Biasini, G. M. Bilei, D. Ciangottini, L. Fanò, P. Lariccia, G. Mantovani, M. Menichelli, F. Romeo, A. Saha, A. Santocchia, A. Spiezia, K. Androsov, P. Azzurri, G. Bagliesi, J. Bernardini, T. Boccali, G. Broccolo, R. Castaldi, M. A. Ciocci, R. Dell’Orso, S. Donato, G. Fedi, F. Fiori, L. Foà, A. Giassi, M. T. Grippo, F. Ligabue, T. Lomtadze, L. Martini, A. Messineo, C. S. Moon, F. Palla, A. Rizzi, A. Savoy-Navarro, A. T. Serban, P. Spagnolo, P. Squillacioti, R. Tenchini, G. Tonelli, A. Venturi, P. G. Verdini, C. Vernieri, L. Barone, F. Cavallari, D. Del Re, M. Diemoz, M. Grassi, C. Jorda, E. Longo, F. Margaroli, P. Meridiani, F. Micheli, S. Nourbakhsh, G. Organtini, R. Paramatti, S. Rahatlou, C. Rovelli, F. Santanastasio, L. Soffi, P. Traczyk, N. Amapane, R. Arcidiacono, S. Argiro, M. Arneodo, R. Bellan, C. Biino, N. Cartiglia, S. Casasso, M. Costa, A. Degano, N. Demaria, L. Finco, C. Mariotti, S. Maselli, E. Migliore, V. Monaco, M. Musich, M. M. Obertino, G. Ortona, L. Pacher, N. Pastrone, M. Pelliccioni, G. L. Pinna Angioni, A. Potenza, A. Romero, M. Ruspa, R. Sacchi, A. Solano, A. Staiano, U. Tamponi, S. Belforte, V. Candelise, M. Casarsa, F. Cossutti, G. DellaRicca, B. Gobbo, C. La Licata, M. Marone, D. Montanino, A. Schizzi, T. Umer, A. Zanetti, S. Chang, T. A. Kropivnitskaya, S. K. Nam, D. H. Kim, G. N. Kim, M. S. Kim, M. S. Kim, D. J. Kong, S. Lee, Y. D. Oh, H. Park, A. Sakharov, D. C. Son, J. Y. Kim, S. Song, S. Choi, D. Gyun, B. Hong, M. Jo, H. Kim, Y. Kim, B. Lee, K. S. Lee, S. K. Park, Y. Roh, M. Choi, J. H. Kim, I. C. Park, S. Park, G. Ryu, M. S. Ryu, Y. Choi, Y. K. Choi, J. Goh, E. Kwon, J. Lee, H. Seo, I. Yu, A. Juodagalvis, J. R. Komaragiri, H. Castilla-Valdez, E. De La Cruz-Burelo, I. Heredia-de LaCruz, R. Lopez-Fernandez, A. Sanchez-Hernandez, S. Carrillo Moreno, F. Vazquez Valencia, I. Pedraza, H. A. Salazar Ibarguen, E. Casimiro Linares, A. Morelos Pineda, D. Krofcheck, P. H. Butler, S. Reucroft, A. Ahmad, M. Ahmad, Q. Hassan, H. R. Hoorani, S. Khalid, W. A. Khan, T. Khurshid, M. A. Shah, M. Shoaib, H. Bialkowska, M. Bluj, B. Boimska, T. Frueboes, M. Górski, M. Kazana, K. Nawrocki, K. Romanowska-Rybinska, M. Szleper, P. Zalewski, G. Brona, K. Bunkowski, M. Cwiok, W. Dominik, K. Doroba, A. Kalinowski, M. Konecki, J. Krolikowski, M. Misiura, M. Olszewski, W. Wolszczak, P. Bargassa, C. Beir ao Da Cruz ESilva, P. Faccioli, P. G. Ferreira Parracho, M. Gallinaro, F. Nguyen, J. Rodrigues Antunes, J. Seixas, J. Varela, P. Vischia, M. Gavrilenko, I. Golutvin, I. Gorbunov, A. Kamenev, V. Karjavin, V. Konoplyanikov, A. Lanev, A. Malakhov, V. Matveev, P. Moisenz, V. Palichik, V. Perelygin, M. Savina, S. Shmatov, S. Shulha, N. Skatchkov, V. Smirnov, A. Zarubin, V. Golovtsov, Y. Ivanov, V. Kim, P. Levchenko, V. Murzin, V. Oreshkin, I. Smirnov, V. Sulimov, L. Uvarov, S. Vavilov, A. Vorobyev, An. Vorobyev, Yu. Andreev, A. Dermenev, S. Gninenko, N. Golubev, M. Kirsanov, N. Krasnikov, A. Pashenkov, D. Tlisov, A. Toropin, V. Epshteyn, V. Gavrilov, N. Lychkovskaya, V. Popov, G. Safronov, S. Semenov, A. Spiridonov, V. Stolin, E. Vlasov, A. Zhokin, V. Andreev, M. Azarkin, I. Dremin, M. Kirakosyan, A. Leonidov, G. Mesyats, S. V. Rusakov, A. Vinogradov, A. Belyaev, E. Boos, M. Dubinin, L. Dudko, A. Ershov, A. Gribushin, V. Klyukhin, O. Kodolova, I. Lokhtin, S. Obraztsov, S. Petrushanko, V. Savrin, A. Snigirev, I. Azhgirey, I. Bayshev, S. Bitioukov, V. Kachanov, A. Kalinin, D. Konstantinov, V. Krychkine, V. Petrov, R. Ryutin, A. Sobol, L. Tourtchanovitch, S. Troshin, N. Tyurin, A. Uzunian, A. Volkov, P. Adzic, M. Dordevic, M. Ekmedzic, J. Milosevic, J. Alcaraz Maestre, C. Battilana, E. Calvo, M. Cerrada, M. Chamizo Llatas, N. Colino, B. De La Cruz, A. Delgado Peris, D. Domínguez Vázquez, A. Escalante Del Valle, C. Fernandez Bedoya, J. P. Fernández Ramos, J. Flix, M. C. Fouz, P. Garcia-Abia, O. Gonzalez Lopez, S. Goy Lopez, J. M. Hernandez, M. I. Josa, G. Merino, E. Navarro De Martino, A. Pérez-Calero Yzquierdo, J. Puerta Pelayo, A. QuintarioOlmeda, I. Redondo, L. Romero, M. S. Soares, C. Albajar, J. F. de Trocóniz, M. Missiroli, H. Brun, J. Cuevas, J. Fernandez Menendez, S. Folgueras, I. GonzalezCaballero, L. Lloret Iglesias, J. A. Brochero Cifuentes, I. J. Cabrillo, A. Calderon, J. DuarteCampderros, M. Fernandez, G. Gomez, A. Graziano, A. Lopez Virto, J. Marco, R. Marco, C. Martinez Rivero, F. Matorras, F. J. MunozSanchez, J. Piedra Gomez, T. Rodrigo, A. Y. Rodríguez-Marrero, A. Ruiz-Jimeno, L. Scodellaro, I. Vila, R. Vilar Cortabitarte, D. Abbaneo, E. Auffray, G. Auzinger, M. Bachtis, P. Baillon, A. H. Ball, D. Barney, A. Benaglia, J. Bendavid, L. Benhabib, J. F. Benitez, C. Bernet, G. Bianchi, P. Bloch, A. Bocci, A. Bonato, O. Bondu, C. Botta, H. Breuker, T. Camporesi, G. Cerminara, S. Colafranceschi, M. D’Alfonso, D. d’Enterria, A. Dabrowski, A. David, F. De Guio, A. De Roeck, S. De Visscher, M. Dobson, N. Dupont-Sagorin, A. Elliott-Peisert, J. Eugster, G. Franzoni, W. Funk, M. Giffels, D. Gigi, K. Gill, D. Giordano, M. Girone, F. Glege, R. Guida, S. Gundacker, M. Guthoff, J. Hammer, M. Hansen, P. Harris, J. Hegeman, V. Innocente, P. Janot, K. Kousouris, K. Krajczar, P. Lecoq, C. Lourenço, N. Magini, L. Malgeri, M. Mannelli, L. Masetti, F. Meijers, S. Mersi, E. Meschi, F. Moortgat, S. Morovic, M. Mulders, P. Musella, L. Orsini, L. Pape, E. Perez, L. Perrozzi, A. Petrilli, G. Petrucciani, A. Pfeiffer, M. Pierini, M. Pimiä, D. Piparo, M. Plagge, A. Racz, G. Rolandi, M. Rovere, H. Sakulin, C. Schäfer, C. Schwick, S. Sekmen, A. Sharma, P. Siegrist, P. Silva, M. Simon, P. Sphicas, D. Spiga, J. Steggemann, B. Stieger, M. Stoye, D. Treille, A. Tsirou, G. I. Veres, J.R. Vlimant, N. Wardle, H. K. Wöhri, W. D. Zeuner, W. Bertl, K. Deiters, W. Erdmann, R. Horisberger, Q. Ingram, H. C. Kaestli, S. König, D. Kotlinski, U. Langenegger, D. Renker, T. Rohe, F. Bachmair, L. Bäni, L. Bianchini, P. Bortignon, M. A. Buchmann, B. Casal, N. Chanon, A. Deisher, G. Dissertori, M. Dittmar, M. Donegà, M. Dünser, P. Eller, C. Grab, D. Hits, W. Lustermann, B. Mangano, A. C. Marini, P. Martinez Ruiz delArbol, D. Meister, N. Mohr, C. Nägeli, P. Nef, F. Nessi-Tedaldi, F. Pandolfi, F. Pauss, M. Peruzzi, M. Quittnat, L. Rebane, F.J. Ronga, M. Rossini, A. Starodumov, M. Takahashi, K. Theofilatos, R. Wallny, H. A. Weber, C. Amsler, M. F. Canelli, V. Chiochia, A. De Cosa, A. Hinzmann, T. Hreus, M. Ivova Rikova, B. Kilminster, B. MillanMejias, J. Ngadiuba, P. Robmann, H. Snoek, S. Taroni, M. Verzetti, Y. Yang, M. Cardaci, K. H. Chen, C. Ferro, C. M. Kuo, W. Lin, Y. J. Lu, R. Volpe, S. S. Yu, P. Chang, Y. H. Chang, Y. W. Chang, Y. Chao, K. F. Chen, P. H. Chen, C. Dietz, U. Grundler, W.-S. Hou, K. Y. Kao, Y. J. Lei, Y. F. Liu, R.-S. Lu, D. Majumder, E. Petrakou, Y. M. Tzeng, R. Wilken, B. Asavapibhop, N. Srimanobhas, N. Suwonjandee, A. Adiguzel, M. N. Bakirci, S. Cerci, C. Dozen, I. Dumanoglu, E. Eskut, S. Girgis, G. Gokbulut, E. Gurpinar, I. Hos, E. E. Kangal, A. KayisTopaksu, G. Onengut, K. Ozdemir, S. Ozturk, A. Polatoz, K. Sogut, D. Sunar Cerci, B. Tali, H. Topakli, M. Vergili, I. V. Akin, B. Bilin, S. Bilmis, H. Gamsizkan, B. Isildak, G. Karapinar, K. Ocalan, U. E. Surat, M. Yalvac, M. Zeyrek, E. Gülmez, B. Isildak, M. Kaya, O. Kaya, H. Bahtiyar, E. Barlas, K. Cankocak, F. I. Vardarlı, M. Yücel, L. Levchuk, P. Sorokin, J. J. Brooke, E. Clement, D. Cussans, H. Flacher, R. Frazier, J. Goldstein, M. Grimes, G. P. Heath, H. F. Heath, J. Jacob, L. Kreczko, C. Lucas, Z. Meng, D. M. Newbold, S. Paramesvaran, A. Poll, S. Senkin, V. J. Smith, T. Williams, K. W. Bell, A. Belyaev, C. Brew, R. M. Brown, D. J. A. Cockerill, J. A. Coughlan, K. Harder, S. Harper, E. Olaiya, D. Petyt, C. H. Shepherd-Themistocleous, A. Thea, I. R. Tomalin, W. J. Womersley, S. D. Worm, M. Baber, R. Bainbridge, O. Buchmuller, D. Burton, D. Colling, N. Cripps, M. Cutajar, P. Dauncey, G. Davies, M. Della Negra, P. Dunne, W. Ferguson, J. Fulcher, D. Futyan, A. Gilbert, G. Hall, G. Iles, M. Jarvis, G. Karapostoli, M. Kenzie, R. Lane, R. Lucas, L. Lyons, A.-M. Magnan, S. Malik, J. Marrouche, B. Mathias, J. Nash, A. Nikitenko, J. Pela, M. Pesaresi, K. Petridis, D. M. Raymond, S. Rogerson, A. Rose, C. Seez, P. Sharp, A. Tapper, M. VazquezAcosta, T. Virdee, J. E. Cole, P. R. Hobson, A. Khan, P. Kyberd, D. Leggat, D. Leslie, W. Martin, I. D. Reid, P. Symonds, L. Teodorescu, M. Turner, J. Dittmann, K. Hatakeyama, A. Kasmi, H. Liu, T. Scarborough, O. Charaf, S. I. Cooper, C. Henderson, P. Rumerio, A. Avetisyan, T. Bose, C. Fantasia, A. Heister, P. Lawson, C. Richardson, J. Rohlf, D. Sperka, J. St. John, L. Sulak, J. Alimena, S. Bhattacharya, G. Christopher, D. Cutts, Z. Demiragli, A. Ferapontov, A. Garabedian, S. Jabeen, U. Heintz, G. Kukartsev, E. Laird, G. Landsberg, M. Luk, M. Narain, M. Segala, T. Sinthuprasith, T. Speer, J. Swanson, R. Breedon, G. Breto, M. Calderon De La Barca Sanchez, S. Chauhan, M. Chertok, J. Conway, R. Conway, P. T. Cox, R. Erbacher, M. Gardner, W. Ko, R. Lander, T. Miceli, M. Mulhearn, D. Pellett, J. Pilot, F. Ricci-Tam, M. Searle, S. Shalhout, J. Smith, M. Squires, D. Stolp, M. Tripathi, S. Wilbur, R. Yohay, R. Cousins, P. Everaerts, C. Farrell, J. Hauser, M. Ignatenko, G. Rakness, E. Takasugi, V. Valuev, M. Weber, J. Babb, R. Clare, J. Ellison, J. W. Gary, G. Hanson, J. Heilman, P. Jandir, E. Kennedy, F. Lacroix, H. Liu, O. R. Long, A. Luthra, M. Malberti, H. Nguyen, A. Shrinivas, S. Sumowidagdo, S. Wimpenny, W. Andrews, J. G. Branson, G. B. Cerati, S. Cittolin, R. T. D’Agnolo, D. Evans, A. Holzner, R. Kelley, D. Kovalskyi, M. Lebourgeois, J. Letts, I. Macneill, D. Olivito, S. Padhi, C. Palmer, M. Pieri, M. Sani, V. Sharma, S. Simon, E. Sudano, Y. Tu, A. Vartak, C. Welke, F. Würthwein, A. Yagil, J. Yoo, D. Barge, J. Bradmiller-Feld, C. Campagnari, T. Danielson, A. Dishaw, K. Flowers, M. Franco Sevilla, P. Geffert, C. George, F. Golf, L. Gouskos, J. Incandela, C. Justus, N. Mccoll, J. Richman, D. Stuart, W. To, C. West, A. Apresyan, A. Bornheim, J. Bunn, Y. Chen, E. Di Marco, J. Duarte, A. Mott, H. B. Newman, C. Pena, C. Rogan, M. Spiropulu, V. Timciuc, R. Wilkinson, S. Xie, R. Y. Zhu, V. Azzolini, A. Calamba, T. Ferguson, Y. Iiyama, M. Paulini, J. Russ, H. Vogel, I. Vorobiev, J. P. Cumalat, B. R. Drell, W. T. Ford, A. Gaz, E. LuiggiLopez, U. Nauenberg, J. G. Smith, K. Stenson, K. A. Ulmer, S. R. Wagner, J. Alexander, A. Chatterjee, J. Chu, S. Dittmer, N. Eggert, W. Hopkins, B. Kreis, N. Mirman, G. Nicolas Kaufman, J. R. Patterson, A. Ryd, E. Salvati, L. Skinnari, W. Sun, W. D. Teo, J. Thom, J. Thompson, J. Tucker, Y. Weng, L. Winstrom, P. Wittich, D. Winn, S. Abdullin, M. Albrow, J. Anderson, G. Apollinari, L. A. T. Bauerdick, A. Beretvas, J. Berryhill, P. C. Bhat, K. Burkett, J. N. Butler, H. W. K. Cheung, F. Chlebana, S. Cihangir, V. D. Elvira, I. Fisk, J. Freeman, Y. Gao, E. Gottschalk, L. Gray, D. Green, S. Grünendahl, O. Gutsche, J. Hanlon, D. Hare, R. M. Harris, J. Hirschauer, B. Hooberman, S. Jindariani, M. Johnson, U. Joshi, K. Kaadze, B. Klima, S. Kwan, J. Linacre, D. Lincoln, R. Lipton, T. Liu, J. Lykken, K. Maeshima, J. M. Marraffino, V. I. Martinez Outschoorn, S. Maruyama, D. Mason, P. McBride, K. Mishra, S. Mrenna, Y. Musienko, S. Nahn, C. Newman-Holmes, V. O’Dell, O. Prokofyev, E. Sexton-Kennedy, S. Sharma, A. Soha, W. J. Spalding, L. Spiegel, L. Taylor, S. Tkaczyk, N. V. Tran, L. Uplegger, E. W. Vaandering, R. Vidal, A. Whitbeck, J. Whitmore, F. Yang, D. Acosta, P. Avery, D. Bourilkov, M. Carver, T. Cheng, D. Curry, S. Das, M. De Gruttola, G. P. Di Giovanni, R. D. Field, M. Fisher, I. K. Furic, J. Hugon, J. Konigsberg, A. Korytov, T. Kypreos, J. F. Low, K. Matchev, P. Milenovic, G. Mitselmakher, L. Muniz, A. Rinkevicius, L. Shchutska, N. Skhirtladze, M. Snowball, J. Yelton, M. Zakaria, V. Gaultney, S. Hewamanage, S. Linn, P. Markowitz, G. Martinez, J. L. Rodriguez, T. Adams, A. Askew, J. Bochenek, B. Diamond, J. Haas, S. Hagopian, V. Hagopian, K. F. Johnson, H. Prosper, V. Veeraraghavan, M. Weinberg, M. M. Baarmand, M. Hohlmann, H. Kalakhety, F. Yumiceva, M. R. Adams, L. Apanasevich, V. E. Bazterra, D. Berry, R. R. Betts, I. Bucinskaite, R. Cavanaugh, O. Evdokimov, L. Gauthier, C. E. Gerber, D. J. Hofman, S. Khalatyan, P. Kurt, D. H. Moon, C. O’Brien, C. Silkworth, P. Turner, N. Varelas, E. A. Albayrak, B. Bilki, W. Clarida, K. Dilsiz, F. Duru, M. Haytmyradov, J.-P. Merlo, H. Mermerkaya, A. Mestvirishvili, A. Moeller, J. Nachtman, H. Ogul, Y. Onel, F. Ozok, A. Penzo, R. Rahmat, S. Sen, P. Tan, E. Tiras, J. Wetzel, T. Yetkin, K. Yi, B. A. Barnett, B. Blumenfeld, S. Bolognesi, D. Fehling, A. V. Gritsan, P. Maksimovic, C. Martin, M. Swartz, P. Baringer, A. Bean, G. Benelli, C. Bruner, J. Gray, R. P. Kenny, M. Murray, D. Noonan, S. Sanders, J. Sekaric, R. Stringer, Q. Wang, J. S. Wood, A. F. Barfuss, I. Chakaberia, A. Ivanov, S. Khalil, M. Makouski, Y. Maravin, L. K. Saini, S. Shrestha, I. Svintradze, J. Gronberg, D. Lange, F. Rebassoo, D. Wright, A. Baden, B. Calvert, S. C. Eno, J. A. Gomez, N. J. Hadley, R. G. Kellogg, T. Kolberg, Y. Lu, M. Marionneau, A. C. Mignerey, K. Pedro, A. Skuja, M. B. Tonjes, S. C. Tonwar, A. Apyan, R. Barbieri, G. Bauer, W. Busza, I. A. Cali, M. Chan, L. Di Matteo, V. Dutta, G. Gomez Ceballos, M. Goncharov, D. Gulhan, M. Klute, Y. S. Lai, Y.-J. Lee, A. Levin, P. D. Luckey, T. Ma, C. Paus, D. Ralph, C. Roland, G. Roland, G. S. F. Stephans, F. Stöckli, K. Sumorok, D. Velicanu, J. Veverka, B. Wyslouch, M. Yang, M. Zanetti, V. Zhukova, B. Dahmes, A. De Benedetti, A. Gude, S. C. Kao, K. Klapoetke, Y. Kubota, J. Mans, N. Pastika, R. Rusack, A. Singovsky, N. Tambe, J. Turkewitz, J. G. Acosta, S. Oliveros, E. Avdeeva, K. Bloom, S. Bose, D. R. Claes, A. Dominguez, R. Gonzalez Suarez, J. Keller, D. Knowlton, I. Kravchenko, J. Lazo-Flores, S. Malik, F. Meier, G. R. Snow, J. Dolen, A. Godshalk, I. Iashvili, A. Kharchilava, A. Kumar, S. Rappoccio, G. Alverson, E. Barberis, D. Baumgartel, M. Chasco, J. Haley, A. Massironi, D. M. Morse, D. Nash, T. Orimoto, D. Trocino, D. Wood, J. Zhang, K. A. Hahn, A. Kubik, N. Mucia, N. Odell, B. Pollack, A. Pozdnyakov, M. Schmitt, S. Stoynev, K. Sung, M. Velasco, S. Won, A. Brinkerhoff, K. M. Chan, A. Drozdetskiy, M. Hildreth, C. Jessop, D. J. Karmgard, N. Kellams, K. Lannon, W. Luo, S. Lynch, N. Marinelli, T. Pearson, M. Planer, R. Ruchti, N. Valls, M. Wayne, M. Wolf, A. Woodard, L. Antonelli, J. Brinson, B. Bylsma, L. S. Durkin, S. Flowers, C. Hill, R. Hughes, K. Kotov, T. Y. Ling, D. Puigh, M. Rodenburg, G. Smith, C. Vuosalo, B. L. Winer, H. Wolfe, H. W. Wulsin, E. Berry, O. Driga, P. Elmer, P. Hebda, A. Hunt, S. A. Koay, P. Lujan, D. Marlow, T. Medvedeva, M. Mooney, J. Olsen, P. Piroué, X. Quan, H. Saka, D. Stickland, C. Tully, J. S. Werner, S. C. Zenz, A. Zuranski, E. Brownson, H. Mendez, J. E. Ramirez Vargas, E. Alagoz, V. E. Barnes, D. Benedetti, G. Bolla, D. Bortoletto, M. De Mattia, A. Everett, Z. Hu, M. K. Jha, M. Jones, K. Jung, M. Kress, N. Leonardo, D. Lopes Pegna, V. Maroussov, P. Merkel, D. H. Miller, N. Neumeister, B. C. Radburn-Smith, X. Shi, I. Shipsey, D. Silvers, A. Svyatkovskiy, F. Wang, W. Xie, L. Xu, H. D. Yoo, J. Zablocki, Y. Zheng, N. Parashar, J. Stupak, A. Adair, B. Akgun, K. M. Ecklund, F. J. M. Geurts, W. Li, B. Michlin, B. P. Padley, R. Redjimi, J. Roberts, J. Zabel, B. Betchart, A. Bodek, R. Covarelli, P. deBarbaro, R. Demina, Y. Eshaq, T. Ferbel, A. Garcia-Bellido, P. Goldenzweig, J. Han, A. Harel, A. Khukhunaishvili, D. C. Miner, G. Petrillo, D. Vishnevskiy, A. Bhatti, R. Ciesielski, L. Demortier, K. Goulianos, G. Lungu, C. Mesropian, S. Arora, A. Barker, J. P. Chou, C. Contreras-Campana, E. Contreras-Campana, D. Duggan, D. Ferencek, Y. Gershtein, R. Gray, E. Halkiadakis, D. Hidas, A. Lath, S. Panwalkar, M. Park, R. Patel, V. Rekovic, S. Salur, S. Schnetzer, C. Seitz, S. Somalwar, R. Stone, S. Thomas, P. Thomassen, M. Walker, K. Rose, S. Spanier, A. York, O. Bouhali, R. Eusebi, W. Flanagan, J. Gilmore, T. Kamon, V. Khotilovich, V. Krutelyov, R. Montalvo, I. Osipenkov, Y. Pakhotin, A. Perloff, J. Roe, A. Rose, A. Safonov, T. Sakuma, I. Suarez, A. Tatarinov, N. Akchurin, C. Cowden, J. Damgov, C. Dragoiu, P. R. Dudero, J. Faulkner, K. Kovitanggoon, S. Kunori, S. W. Lee, T. Libeiro, I. Volobouev, E. Appelt, A. G. Delannoy, S. Greene, A. Gurrola, W. Johns, C. Maguire, Y. Mao, A. Melo, M. Sharma, P. Sheldon, B. Snook, S. Tuo, J. Velkovska, M. W. Arenton, S. Boutle, B. Cox, B. Francis, J. Goodell, R. Hirosky, A. Ledovskoy, H. Li, C. Lin, C. Neu, J. Wood, S. Gollapinni, R. Harr, P. E. Karchin, C. Kottachchi Kankanamge Don, P. Lamichhane, J. Sturdy, D. A. Belknap, D. Carlsmith, M. Cepeda, S. Dasu, S. Duric, E. Friis, R. Hall-Wilton, M. Herndon, A. Hervé, P. Klabbers, A. Lanaro, C. Lazaridis, A. Levine, R. Loveless, A. Mohapatra, I. Ojalvo, T. Perry, G. A. Pierro, G. Polese, I. Ross, T. Sarangi, A. Savin, W. H. Smith, N. Woods, [Authorinst]CMS Collaboration

**Affiliations:** Yerevan Physics Institute, Yerevan, Armenia; Institut für Hochenergiephysik der OeAW, Vienna, Austria; National Centre for Particle and High Energy Physics, Minsk, Belarus; Universiteit Antwerpen, Antwerp, Belgium; Vrije Universiteit Brussel, Brussels, Belgium; Université Libre de Bruxelles, Brussels, Belgium; Ghent University, Ghent, Belgium; Université Catholique de Louvain, Louvain-la-Neuve, Belgium; Université de Mons, Mons, Belgium; Centro Brasileiro de Pesquisas Fisicas, Rio de Janeiro, Brazil; Universidade do Estado do Rio de Janeiro, Rio de Janeiro, Brazil; Universidade Estadual Paulista, Universidade Federal do ABC, São Paulo, Brazil; Institute for Nuclear Research and Nuclear Energy, Sofia, Bulgaria; University of Sofia, Sofia, Bulgaria; Institute of High Energy Physics, Beijing, China; State Key Laboratory of Nuclear Physics and Technology, Peking University, Beijing, China; Universidad de Los Andes, Bogotá, Colombia; Faculty of Electrical Engineering, Mechanical Engineering and Naval Architecture, University of Split, Split, Croatia; Faculty of Science, University of Split, Split, Croatia; Institute Rudjer Boskovic, Zagreb, Croatia; University of Cyprus, Nicosia, Cyprus; Charles University, Prague, Czech Republic; Academy of Scientific Research and Technology of the Arab Republic of Egypt, Egyptian Network of High Energy Physics, Cairo, Egypt; National Institute of Chemical Physics and Biophysics, Tallinn, Estonia; Department of Physics, University of Helsinki, Helsinki, Finland; Helsinki Institute of Physics, Helsinki, Finland; Lappeenranta University of Technology, Lappeenranta, Finland; DSM/IRFU, CEA/Saclay, Gif-sur-Yvette, France; Laboratoire Leprince-Ringuet, Ecole Polytechnique, IN2P3-CNRS, Palaiseau, France; Institut Pluridisciplinaire Hubert Curien, Université de Strasbourg, Université de Haute Alsace Mulhouse, CNRS/IN2P3, Strasbourg, France; Centre de Calcul de l’Institut National de Physique Nucleaire et de Physique des Particules, CNRS/IN2P3, Villeurbanne, France; Institut de Physique Nucléaire de Lyon, Université de Lyon, Université Claude Bernard Lyon 1, CNRS-IN2P3, Villeurbanne, France; E. Andronikashvili Institute of Physics, Academy of Science, Tbilisi, Georgia; I. Physikalisches Institut, RWTH Aachen University, Aachen, Germany; III. Physikalisches Institut A, RWTH Aachen University, Aachen, Germany; III. Physikalisches Institut B, RWTH Aachen University, Aachen, Germany; Deutsches Elektronen-Synchrotron, Hamburg, Germany; University of Hamburg, Hamburg, Germany; Institut für Experimentelle Kernphysik, Karlsruhe, Germany; Institute of Nuclear and Particle Physics (INPP), NCSR Demokritos, Aghia Paraskevi, Greece; University of Athens, Athens, Greece; University of Ioánnina, Ioannina, Greece; Wigner Research Centre for Physics, Budapest, Hungary; Institute of Nuclear Research ATOMKI, Debrecen, Hungary; University of Debrecen, Debrecen, Hungary; National Institute of Science Education and Research, Bhubaneswar, India; Panjab University, Chandigarh, India; University of Delhi, Delhi, India; Saha Institute of Nuclear Physics, Kolkata, India; Bhabha Atomic Research Centre, Mumbai, India; Tata Institute of Fundamental Research, Mumbai, India; Institute for Research in Fundamental Sciences (IPM), Tehran, Iran; University College Dublin, Dublin, Ireland; INFN Sezione di Bari, Università di Bari, Politecnico di Bari, Bari, Italy; INFN Sezione di Bologna, Università di Bologna, Bologna, Italy; INFN Sezione di Catania, Università di Catania, CSFNSM, Catania, Italy; INFN Sezione di Firenze, Università di Firenze, Florence, Italy; INFN Laboratori Nazionali di Frascati, Frascati, Italy; INFN Sezione di Genova, Università di Genova, Genoa, Italy; INFN Sezione di Milano-Bicocca, Università di Milano-Bicocca, Milan, Italy; INFN Sezione di Napoli, Università di Napoli ’Federico II’, Università della Basilicata (Potenza), Università G. Marconi (Roma), Naples, Italy; INFN Sezione di Padova, Università di Padova, Università di Trento (Trento), Padua, Italy; INFN Sezione di Pavia, Università di Pavia, Pavia, Italy; INFN Sezione di Perugia, Università di Perugia, Perugia, Italy; INFN Sezione di Pisa, Università di Pisa, Scuola Normale Superiore di Pisa, Pisa, Italy; INFN Sezione di Roma, Università di Roma, Rome, Italy; INFN Sezione di Torino, Università di Torino, Università del Piemonte Orientale (Novara), Turin, Italy; INFN Sezione di Trieste, Università di Trieste, Trieste, Italy; Kangwon National University, Chunchon, Korea; Kyungpook National University, Taegu, Korea; Institute for Universe and Elementary Particles, Chonnam National University, Kwangju, Korea; Korea University, Seoul, Korea; University of Seoul, Seoul, Korea; Sungkyunkwan University, Suwon, Korea; Vilnius University, Vilnius, Lithuania; National Centre for Particle Physics, Universiti Malaya, Kuala Lumpur, Malaysia; Centro de Investigacion y de Estudios Avanzados del IPN, Mexico City, Mexico; Universidad Iberoamericana, Mexico City, Mexico; Benemerita Universidad Autonoma de Puebla, Puebla, Mexico; Universidad Autónoma de San Luis Potosí, San Luis Potosí, Mexico; University of Auckland, Auckland, New Zealand; University of Canterbury, Christchurch, New Zealand; National Centre for Physics, Quaid-I-Azam University, Islamabad, Pakistan; National Centre for Nuclear Research, Swierk, Poland; Institute of Experimental Physics, Faculty of Physics, University of Warsaw, Warsaw, Poland; Laboratório de Instrumentação e Física Experimental de Partículas, Lisbon, Portugal; Joint Institute for Nuclear Research, Dubna, Russia; Petersburg Nuclear Physics Institute, Gatchina, St. Petersburg, Russia; Institute for Nuclear Research, Moscow, Russia; Institute for Theoretical and Experimental Physics, Moscow, Russia; P. N. Lebedev Physical Institute, Moscow, Russia; Skobeltsyn Institute of Nuclear Physics, Lomonosov Moscow State University, Moscow, Russia; State Research Center of Russian Federation, Institute for High Energy Physics, Protvino, Russia; Faculty of Physics and Vinca Institute of Nuclear Sciences, University of Belgrade, Belgrade, Serbia; Centro de Investigaciones Energéticas Medioambientales y Tecnológicas (CIEMAT), Madrid, Spain; Universidad Autónoma de Madrid, Madrid, Spain; Universidad de Oviedo, Oviedo, Spain; Instituto de Física de Cantabria (IFCA), CSIC-Universidad de Cantabria, Santander, Spain; CERN, European Organization for Nuclear Research, Geneva, Switzerland; Paul Scherrer Institut, Villigen, Switzerland; Institute for Particle Physics, ETH Zurich, Zurich, Switzerland; Universität Zürich, Zurich, Switzerland; National Central University, Chung-Li, Taiwan; National Taiwan University (NTU), Taipei, Taiwan; Department of Physics, Faculty of Science, Chulalongkorn University, Bangkok, Thailand; Cukurova University, Adana, Turkey; Physics Department, Middle East Technical University, Ankara, Turkey; Bogazici University, Istanbul, Turkey; Istanbul Technical University, Istanbul, Turkey; National Scientific Center, Kharkov Institute of Physics and Technology, Kharkov, Ukraine; University of Bristol, Bristol, UK; Rutherford Appleton Laboratory, Didcot, UK; Imperial College, London, UK; Brunel University, Uxbridge, UK; Baylor University, Waco, USA; The University of Alabama, Tuscaloosa, USA; Boston University, Boston, USA; Brown University, Providence, USA; University of California, Davis, Davis, USA; University of California, Los Angeles, USA; University of California, Riverside, Riverside, USA; University of California, San Diego, La Jolla, USA; University of California, Santa Barbara, Santa Barbara, USA; California Institute of Technology, Pasadena, USA; Carnegie Mellon University, Pittsburgh, USA; University of Colorado at Boulder, Boulder, USA; Cornell University, Ithaca, USA; Fairfield University, Fairfield, USA; Fermi National Accelerator Laboratory, Batavia, USA; University of Florida, Gainesville, USA; Florida International University, Miami, USA; Florida State University, Tallahassee, USA; Florida Institute of Technology, Melbourne, USA; University of Illinois at Chicago (UIC), Chicago, USA; The University of Iowa, Iowa City, USA; Johns Hopkins University, Baltimore, USA; The University of Kansas, Lawrence, USA; Kansas State University, Manhattan, USA; Lawrence Livermore National Laboratory, Livermore, USA; University of Maryland, College Park, USA; Massachusetts Institute of Technology, Cambridge, USA; University of Minnesota, Minneapolis, USA; University of Mississippi, Oxford, USA; University of Nebraska-Lincoln, Lincoln, USA; State University of New York at Buffalo, Buffalo, USA; Northeastern University, Boston, USA; Northwestern University, Evanston, USA; University of Notre Dame, Notre Dame, USA; The Ohio State University, Columbus, USA; Princeton University, Princeton, USA; University of Puerto Rico, Mayagüez, USA; Purdue University, West Lafayette, USA; Purdue University Calumet, Hammond, USA; Rice University, Houston, USA; University of Rochester, Rochester, USA; The Rockefeller University, New York, USA; Rutgers, The State University of New Jersey, Piscataway, USA; University of Tennessee, Knoxville, USA; Texas A&M University, College Station, USA; Texas Tech University, Lubbock, USA; Vanderbilt University, Nashville, USA; University of Virginia, Charlottesville, USA; Wayne State University, Detroit, USA; University of Wisconsin, Madison, USA

## Abstract

Results are presented from a search for particle dark matter (DM), extra dimensions, and unparticles using events containing a jet and an imbalance in transverse momentum. The data were collected by the CMS detector in proton–proton collisions at the LHC and correspond to an integrated luminosity of 19.7$\,\text {fb}^\text {-1}$at a centre-of-mass energy of 8$\,{\mathrm{TeV}}\,$. The number of observed events is found to be consistent with the standard model prediction. Limits are placed on the DM-nucleon scattering cross section as a function of the DM particle mass for spin-dependent and spin-independent interactions. Limits are also placed on the scale parameter $M_\mathrm {D}$ in the Arkani-Hamed, Dimopoulos, and Dvali (ADD) model of large extra dimensions, and on the unparticle model parameter ${{\Lambda _\mathrm{U}}}$. The constraints on ADD models and unparticles are the most stringent limits in this channel and those on the DM-nucleon scattering cross section are an improvement over previous collider results.

## Introduction

This paper describes a search for new physics using the signature of a hadronic jet and an imbalance in transverse energy resulting from undetected particles. We use the term “monojet” to describe events with this topology. Such events can be produced in new physics scenarios, including particle dark matter (DM) production, large extra dimensions, and unparticles. The data sample corresponds to an integrated luminosity of 19.7$\,\text {fb}^\text {-1}$collected by the CMS experiment in proton–proton collisions provided by the CERN LHC at a centre-of-mass energy of 8$\,{\mathrm{TeV}}\,$.

Particle dark matter has been proposed to explain numerous astrophysical measurements, such as the rotation curves of galaxies and gravitational lensing [1, 2]. Popular models of particle dark matter hypothesize the existence of non-relativistic particles that interact weakly with the standard model (SM) particles. These are known as weakly interacting massive particles (WIMPs). Such models are consistent with the thermal relic abundance for dark matter [3, 4] if the WIMPs have weak-scale masses and if their interaction cross section with baryonic matter is of the order of electroweak cross sections. Some new physics scenarios postulated to explain the hierarchy problem also predict the existence of WIMPs [5].

Since WIMPs are weakly interacting and neutral, they are not expected to produce any discernible signal in the LHC detectors. Like neutrinos, they remain undetected and their presence in an event must be inferred from an imbalance of the total momentum of all reconstructed particles in the plane transverse to the beam axis. The magnitude of such an imbalance is referred to as missing transverse energy, denoted by $E_{\mathrm {T}}^{\text {miss}}$. The monojet signature can be used to search for the pair production of WIMPs in association with a jet from initial-state radiation (ISR), which is used to tag or trigger the event.

**Fig. 1:**
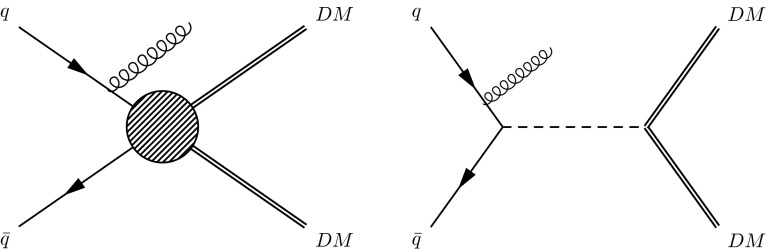
Feynman diagrams for the pair production of DM particles for the case of a contact interaction (*left*) and the exchange of a mediator (*right*)

In this Letter, we investigate two scenarios for producing dark matter particles that have been extensively discussed [6–9]. In the first case, we assume that the mediator responsible for coupling of the SM and DM particles is heavier ($\gtrsim$few $\text {TeV}$) than the typical energy transfer at the LHC. We can thus assume the interaction to be a contact interaction and work within the framework of an effective field theory. In the second case, we consider the scenario in which the mediator is light enough to be produced at the LHC. Figure 1 shows Feynman diagrams leading to the pair production of DM particles for the case of a contact interaction and the exchange of a mediator.

We study interactions that are vector, axial-vector, and scalar, as described in [6, 9], for a Dirac fermion DM particle ($\chi$). The results are not expected to be greatly altered if the DM particle is a Majorana fermion, except that certain interactions are not allowed. Results from previous searches in the monojet channel have been used to set limits on the DM-nucleon scattering cross section as a function of the DM mass [10–12].

The Arkani-Hamed, Dimopoulos, and Dvali (ADD) model [13–17] of large extra dimensions mitigates the hierarchy problem [18] by introducing a number $\delta$ of extra dimensions. In the simplest scenario, these are compactified over a multidimensional torus with radii $R$. Gravity is free to propagate into the extra dimensions, while SM particles and interactions are confined to ordinary space–time. The strength of the gravitational force is thus diluted in $3+1$ dimensional space–time, explaining its apparent weakness in comparison to the other fundamental forces. The fundamental Planck scale in $3+\delta$ spatial dimensions, ${M_\mathrm {D}}$, is related to the apparent Planck scale in 3 dimensions, ${M_\mathrm {Pl}}$ as ${M_\mathrm {Pl}}^{2} = 8\pi {M_\mathrm {D}}^{(\delta +2)}R^{\delta }$ [16]. The increased phase space available in the extra dimensions is expected to enhance the production of gravitons, which are weakly interacting and escape undetected, their presence must therefore be inferred by detecting $E_{\mathrm {T}}^{\text {miss}}$. When produced in association with a jet, this gives rise to the monojet signal. Previous searches for large extra dimensions in monophoton and monojet channels have yielded no evidence of new physics [11, 12, 19–25].

**Fig. 2:**
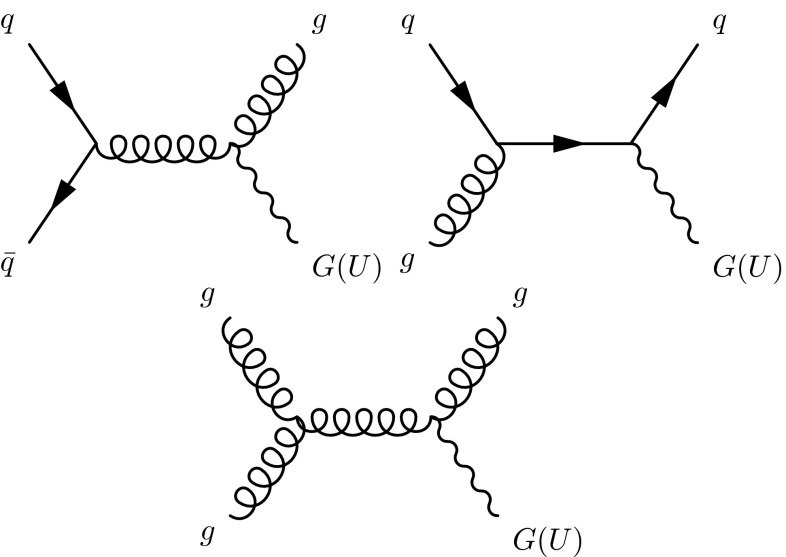
Feynman diagrams for the production of a graviton (*G*) or unparticles (*U*) in association with a jet

Unparticle models [26] postulate the existence of a scale-invariant (conformal) sector, indicating new physics that cannot be described using particles. This conformal sector is connected to the SM at a high mass scale ${{\Lambda _\mathrm{U}}}$. In the low-energy limit, with scale dimension $d_{u}$, events appear to correspond to the production of a non-integer number $d_{u}$ of invisible particles. Assuming these are sufficiently long-lived to decay outside of the detector, they are undetected and so give rise to $E_{\mathrm {T}}^{\text {miss}}$. If ${{\Lambda _\mathrm{U}}}$ is assumed to be of order$\,{\mathrm{TeV}}\,$, the effects of unparticles can be studied in the context of an effective field theory at the LHC. Previous searches for unparticles at CMS [24] have yielded no evidence of new physics. Figure 2 shows Feynman diagrams for some of the processes leading to the production of a graviton or unparticle in association with a jet.

## The CMS detector and event reconstruction

The CMS apparatus features a superconducting solenoid, 12.5$\text {\,m}$ long with an internal diameter of 6$\text {\,m}$, providing a uniform magnetic field of 3.8$\text {\,T}$. Within the field volume are a silicon pixel and strip tracker, a crystal electromagnetic calorimeter and a brass/scintillator hadron calorimeter. The momentum resolution for reconstructed tracks in the central region is about 1.5 % for non-isolated particles with transverse momenta ($p_{\mathrm {T}}$) between 1 and 10$\,\text {GeV}$ and 2.8 % for isolated particles with $p_{\mathrm {T}}$ of 100$\,\text {GeV}$. The calorimeter system surrounds the tracker and consists of a scintillating lead tungstate crystal electromagnetic calorimeter and a brass/scintillator hadron calorimeter with coverage up to $|\eta |=3$. The quartz/steel forward hadron calorimeters extend the calorimetry coverage up to $|\eta |=5$.

A system of gas-ionization muon detectors embedded in the steel flux-return yoke of the solenoid allows reconstruction and identification of muons in the $|\eta | < 2.4$ region. Events are recorded using a two-level trigger system. A more detailed description of the CMS detector and the trigger system can be found in [27].

Offline, particle candidates are individually identified using a particle-flow reconstruction [28, 29]. This algorithm reconstructs each particle produced in a collision by combining information from the tracker, the calorimeters, and the muon system, and identifies them as either a charged hadron, neutral hadron, photon, muon, or electron. The candidate particles are then clustered into jets using the anti-$k_{\mathrm {T}}$ algorithm [30] with a distance parameter of 0.5. The energy resolution for jets is 15 % at $p_{\mathrm {T}}$ of 10$\,\text {GeV}$, 8 % at $p_{\mathrm {T}}$ of 100$\,\text {GeV}$, and 4 % at $p_{\mathrm {T}}$ of 1 $\,{\mathrm{TeV}}\,$ [31]. Corrections are applied to the jet four-momenta as a function of the jet $p_{\mathrm {T}}$ and $\eta$ to account for residual effects of non-uniform detector response [32]. Contributions from multiple proton–proton collisions overlapping with the event of interest (pileup) are mitigated by discarding charged particles not associated with the primary vertex and accounting for the effects from neutral particles [33]. The $E_{\mathrm {T}}^{\text {miss}}$ in this analysis is defined as the magnitude of the vector sum of the transverse momenta of all particles reconstructed in the event, excluding muons.

## Event selection

Events are collected using two triggers, the first of which has an $$E_{\mathrm {T}}^{\text {miss}}$$ threshold of 120$$\,\text {GeV}$$, where the $$E_{\mathrm {T}}^{\text {miss}}$$ is calculated using calorimeter information only. The second trigger requires a particle-flow jet with $$p_{\mathrm {T}} > 80$$$$\,\text {GeV}$$ and $$E_{\mathrm {T}}^{\text {miss}}> 105$$$$\,\text {GeV}$$, where the $$E_{\mathrm {T}}^{\text {miss}}$$ is reconstructed using the particle-flow algorithm and excludes muons. This definition of $$E_{\mathrm {T}}^{\text {miss}}$$ allows the control sample of $$\mathrm {Z}\rightarrow \mathrm {\mu }\mathrm {\mu }$$ events used for estimating the $$\mathrm {Z}\rightarrow \nu \nu $$ background to be collected from the same trigger as the signal sample. The trigger efficiencies are measured to be nearly 100 % for all signal regions. Events are required to have a well-reconstructed primary vertex [34], which is defined as the one with the largest sum of $$p_{\mathrm {T}} ^{2}$$ of all the associated tracks, and is assumed to correspond to the hard scattering process. Instrumental and beam-related backgrounds are suppressed by rejecting events where less than 20 % of the energy of the highest $$p_{\mathrm {T}}$$ jet is carried by charged hadrons, or more than 7 % of this energy is carried by either neutral hadrons or photons. This is very effective in rejecting non-collision backgrounds, which are found to be negligible. The jet with the highest transverse momentum ($$\,\mathrm {j}_1$$) is required to have $$p_{\mathrm {T}} > 110\,\text {GeV} $$ and $$|\eta | < 2.4$$. As signal events typically contain jets from initial state radiation, a second jet ($$\,\mathrm {j}_2$$) with $$p_{\mathrm {T}} $$ above 30$$\,\text {GeV}$$ and $$|\eta | < 4.5$$ is allowed, provided the second jet is separated from the first in azimuth ($$\phi $$) by less than 2.5 radians, $$\Delta \phi (\mathrm {j_1},\mathrm {j_2}) < 2.5$$. This angular requirement suppresses Quantum ChromoDynamics (QCD) dijet events. Events with more than two jets with $$p_{\mathrm {T}} > 30$$$$\,\text {GeV}$$ and $$|\eta | < 4.5$$ are discarded, thereby significantly reducing background from top-quark pair $${{t{\bar{t}}}}$$ and QCD multijet events. Processes producing leptons, such as $$\mathrm {W}$$ and $$\mathrm {Z}$$ production, dibosons, and top-quark decays, are suppressed by rejecting events with well reconstructed and isolated electrons with $$p_{\mathrm {T}} >10\,\text {GeV} $$, reconstructed muons [35] with $$p_{\mathrm {T}} > 10\,\text {GeV} $$ and well-identified [36] hadronically decaying tau leptons with $$p_{\mathrm {T}} > 20\,\text {GeV} $$ and $$|\eta | < 2.3$$. Electrons and muons are considered isolated if the scalar sum of the $$p_{\mathrm {T}} $$ of the charged hadrons, neutral hadrons and photon contributions computed in a cone of radius $$\sqrt{{(\Delta \eta )^{2} + (\Delta \phi )^{2}}} = 0.4$$ about the lepton direction, divided by the electron or muon $$p_{\mathrm {T}} $$, is less than 0.2. The analysis is performed in seven inclusive regions of $$E_{\mathrm {T}}^{\text {miss}}$$: $$E_{\mathrm {T}}^{\text {miss}}>250,$$ 300, 350, 400, 450, 500, 550$$\,\text {GeV}$$.

## Monte Carlo event generation

The DM signal samples are produced using the leading order (LO) matrix element generator MadGraph  [37] interfaced with pythia 6.4.26 [38] with tune Z2* [39] for parton showering and hadronization, and the CTEQ 6L1 [40] parton distribution functions (PDFs). The process of DM pair production is generated with up to two additional partons and a transverse momentum requirement of 80$\,\text {GeV}$ on the partons, with no matching to pythia. Only initial states with gluons and the four lightest quarks are considered and a universal coupling is assumed to all the quarks. The renormalization and factorization scales are set to the sum of $\sqrt{M^{2} + p_{\mathrm {T}} ^{2}}$ for all produced particles, where $M$ is the mass of the particle. For the heavy mediator case, where an effective field theory is assumed, DM particles with masses $M_\chi = 1$, 10, 100, 200, 400, 700, and 1000$\,\text {GeV}$ are generated. For the case of a light mediator, the mediator mass, $M$, is varied from 50$\,\text {GeV}$ all the way up to 10 $\,{\mathrm{TeV}}\,$(to show the effect of the transition to heavy mediators) for DM particle masses of 50 and 500$\,\text {GeV}$. Three separate samples are generated for each value of $M$, with the width, $\Gamma$, of the mediator set to $\Gamma = M/3$, $M/10$, or $M/8\pi$, where $M/3$ and $M/8\pi$ are taken as the extremes of a wide-width and narrow-width mediator, respectively.

The events for the ADD and unparticle models are generated with pythia 8.130 [41, 42] using tune 4C [43] and the CTEQ 6.6M [40] PDFs. This model is an effective theory and holds only for energies well below ${M_\mathrm {D}}$ (${{\Lambda _\mathrm{U}}}$) for the graviton (unparticle). For a parton-parton centre-of-mass energy $\sqrt{\hat{s}}>{M_\mathrm {D}}$ (${{\Lambda _\mathrm{U}}}$), the simulated cross sections of the graviton (unparticle) is suppressed by a factor ${M_\mathrm {D}}^4/\hat{s}^2$ (${{\Lambda _\mathrm{U}}} ^4/\hat{s}^2$) [42]. The renormalization and factorization scales are set to the geometric mean of the squared transverse mass of the outgoing particles.

The MadGraph  [44, 45] generator interfaced with pythia 6.4.26 and the CTEQ 6L1 PDFs is used to produce vector bosons in association with jets ($\mathrm {Z}$ $+$ jets and $\mathrm {W}$ $+$ jets), ${{t{\bar{t}}}}$, or vector bosons in association with photons ($\mathrm {W}\gamma$, $\mathrm {Z}\gamma$). The QCD multijet and diboson ($\mathrm {Z}\mathrm {Z}$, $\mathrm {W}\mathrm {Z}$, $\mathrm {W}\mathrm {W}$) processes are generated with pythia 6.4.26 and CTEQ 6L1 PDFs. Single top-quark events are generated with powheg  [46, 47] interfaced with pythia 6.4.26 and CTEQ 6.6M PDFs. In all cases, pythia 6.4.26 is used with the Z2* tune. All the generated signal and background events are passed through a Geant4  [48, 49] simulation of the CMS detector and reconstructed with the same algorithms as used for collision data. The effect of additional proton–proton interactions in each beam crossing (pileup) is modelled by superimposing minimum bias interactions (obtained using pythia with the Z2* tune) onto the hard interaction, with the multiplicity distribution of primary vertices matching the one observed in data.

## Background estimate

**Fig. 3:**
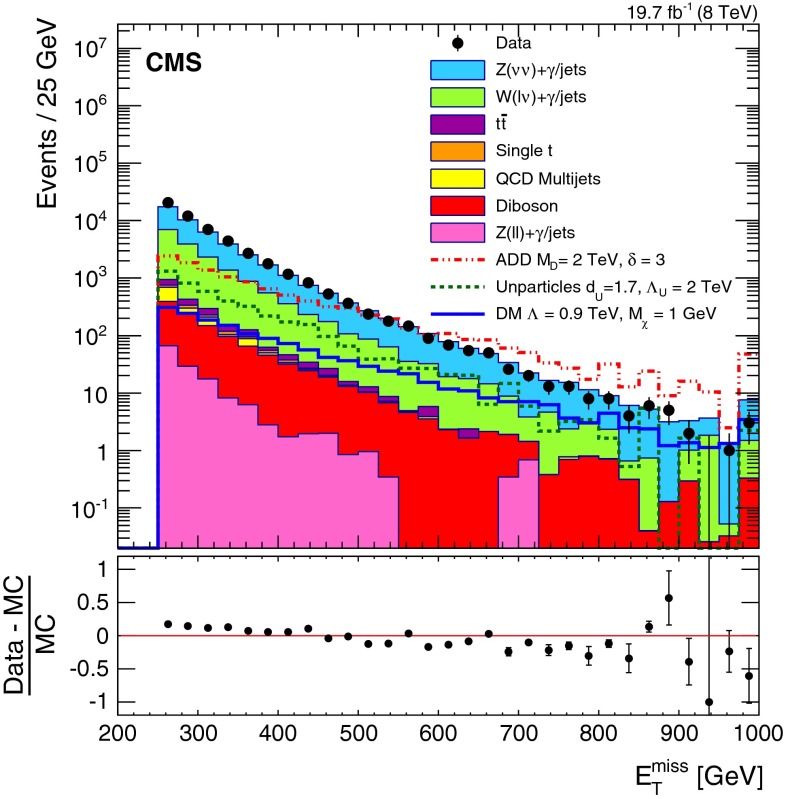
Missing transverse energy $E_{\mathrm {T}}^{\text {miss}}$ after all selections for data and SM backgrounds. The processes contributing to the SM background are from simulation, normalised to the estimation from data using the $E_{\mathrm {T}}^{\text {miss}}$ threshold of 500$\,\text {GeV}$. The *error bars* in the *lower panel* represent the statistical uncertainty. Overflow events are included in the last bin

After the full event selection, there are two dominant backgrounds: Z $+$ jets events with the $\mathrm {Z}$ boson decaying into a pair of neutrinos, denoted $\mathrm {Z}(\nu \nu )$; and $\mathrm {W}$ $+$ jets with the $\mathrm {W}$ boson decaying leptonically, denoted $\mathrm {W}(\ell \nu )\,$ (where $\ell$ stands for a charged lepton, and can be replaced by $\mathrm {e}$, $\mathrm {\mu }$ or $\tau$ to denote specific decays to electron, muon, or tau, respectively). Other background processes include: ${{t{\bar{t}}}}$ production; single top quark, denoted $({{t{\bar{t}}}})$; QCD multijet; diboson processes, including $\mathrm {Z}\mathrm {Z}$, $\mathrm {W}\mathrm {Z}$, and $\mathrm {W}\mathrm {W}$; and $\mathrm {Z}$ $+$ jets events with the $\mathrm {Z}$ boson decaying to charged leptons, denoted $\mathrm {Z}(\ell \ell )$. Together, these other background processes constitute $\approx$4 % of the total. The dominant backgrounds are estimated from data, as described in detail below, whilst others are taken from simulation, and cross-checked with data. Figure 3 shows the $E_{\mathrm {T}}^{\text {miss}}$ distribution of the data and of the expected background, after imposing all the selections described in Sect. 3 and normalised to the estimation from data using the $E_{\mathrm {T}}^{\text {miss}}$ threshold of 500$\,\text {GeV}$.

The background from events containing $$\mathrm {Z}(\nu \nu )$$ decays is estimated from a control data sample of $$\mathrm {Z}(\mu \mu )$$ events, since the kinematic features of the two processes are similar. The control sample is selected by applying the full signal selection, except for the muon veto, and in addition requiring two reconstructed muons with $$p_{\mathrm {T}} > 20$$$$\,\text {GeV}$$ and $$|\eta | < 2.4$$, with at least one muon also passing the isolation requirement. The reconstructed invariant mass is required to be between 60 and 120$$\,\text {GeV}$$. The distribution of $$\mathrm {Z}(\nu \nu )$$ events is estimated from the observed dimuon control sample after correcting for the following: the estimated background in the dimuon sample; differences in muon acceptance and efficiency with respect to neutrinos; and the ratio of branching fractions for the $$\mathrm {Z}$$ decay to a pair of neutrinos, and to a pair of muons ($$R_\mathrm {BF}$$). The acceptance estimate is taken from the fraction of simulated events that pass all signal selection requirements (except muon veto), having two generated muons with $$p_{\mathrm {T}} > 20$$$$\,\text {GeV}$$ and $$|\eta | < 2.4$$ and an invariant mass within the $$\mathrm {Z}$$-boson mass window of 60–120$$\,\text {GeV}$$. The efficiency of the selection, which has the additional requirement that there be at least one isolated muon in the event, is also estimated from simulation. It is corrected to account for differences in the measured muon reconstruction efficiencies in data and simulation. The uncertainty in the $$\mathrm {Z}(\nu \nu )$$ prediction includes both statistical and systematic components. The sources of uncertainty are: (1) the statistical uncertainty in the numbers of $$\mathrm {Z}(\mu \mu )$$ events in the data, (2) uncertainty due to backgrounds contributing to the control sample, (3) uncertainties in the acceptance due to the size of the simulation samples and from PDFs evaluated based on the PDF4LHC [50, 51] recommendations, (4) the uncertainty in the selection efficiency as determined from the difference in measured efficiencies in data and simulation and the size of the simulation samples, and (5) the theoretical uncertainty on the ratio of branching fractions [52]. The backgrounds to the $$\mathrm {Z}(\mu \mu )$$ control sample contribute at the level of 3–5 % across the $$E_{\mathrm {T}}^{\text {miss}}$$ signal regions and are predominantly from diboson and $${{t{\bar{t}}}}$$ processes. These are taken from simulation and a 50 % uncertainty is assigned to them. The dominant source of uncertainty in the high $$E_{\mathrm {T}}^{\text {miss}}$$ regions is the statistical uncertainty in the number of $$\mathrm {Z}(\mu \mu )$$ events, which is 11 % for $$E_{\mathrm {T}}^{\text {miss}}> 500$$$$\,\text {GeV}$$. Table 1 summarizes the statistical and systematic uncertainties.

**Table 1:** Summary of the statistical and systematic contributions to the total uncertainty on the $\mathrm {Z}(\nu \nu )$ background

$E_{\mathrm {T}}^{\text {miss}}$ ($\text {GeV}$ ) $\rightarrow$	$>$250	$>$300	$>$350	$>$400	$>$450	$>$500	$>$550
(1) $\mathrm {Z}(\mu \mu )$ $+$ jets statistical unc.	1.7	2.7	4.0	5.6	7.8	11	16
(2) Background	1.4	1.7	2.1	2.4	2.7	3.2	3.9
(3) Acceptance	2.0	2.1	2.1	2.2	2.3	2.6	2.8
(4) Selection efficiency	2.1	2.2	2.2	2.4	2.7	3.1	3.7
(5) R$_\mathrm {BF}$	2.0	2.0	2.0	2.0	2.0	2.0	2.0
Total uncertainty (%)	5.1	5.6	6.6	7.9	9.9	13	18

The second-largest background arises from $$\mathrm {W}$$ $$+$$ jets events that are not rejected by the lepton veto. This can occur when a lepton (electron or muon) from the W decays (prompt or via leptonic tau decay) fails the identification, isolation or acceptance requirements, or a hadronic tau decay is not identified. The contributions to the signal region from these events are estimated from the $$\mathrm {W}(\mu \nu )\,+$$ jets control sample in data. This sample is selected by applying the full signal selection, except the muon veto, and instead requiring an isolated muon with $$p_{\mathrm {T}} > 20$$$$\,\text {GeV}$$ and $$|\eta | < 2.4$$, and the transverse mass $$M_\mathrm{T}$$ to be between 50 and 100$$\,\text {GeV}$$. Here $$M_\mathrm {T}=\sqrt{2p_{\mathrm {T}} ^{\mu }E_{\mathrm {T}}^{\text {miss}}\left( 1-\cos \Delta \phi \right) }$$, where $$p_{\mathrm {T}} ^{\mathrm {\mu }}$$ is the transverse momentum of the muon and $$\Delta \phi $$ is the azimuthal angle between the muon direction of flight and the negative of the sum of the transverse momenta of all the particles reconstructed in the event.

The observed number of events in the $\mathrm {W}$ control sample is used to find the numbers of $\mathrm {W}(\mu \nu )\,+$ jets events passing the selection steps prior to the lepton veto. The required corrections for background contamination of the control sample, and for the acceptance and efficiency are taken from simulation. Using these correction factors, we estimate the fraction of events containing muons that are not identified, either due to inefficiencies in the reconstruction or because they have trajectories outside the muon system acceptance. This acceptance and the selection efficiency are also taken from simulation. Such events will not be rejected by the lepton veto and so contribute to the background in the signal region.

In addition, there are similar contributions from $\mathrm {W}$ decays to electrons and tau leptons. These contributions are also estimated based on the $\mathrm {W}(\mu \nu )\,+$ jets sample. The ratio of $\mathrm {W}(\ell \nu )\, +$ jets events to $\mathrm {W}(\mu \nu )\,+$ jets events passing the selection steps prior to the lepton veto is taken from simulation, separately for each lepton flavor. The same procedure as that used in the muon case is then applied to obtain the background contribution to the signal region.

The detector acceptances for electrons, muons and tau leptons are obtained from simulation. The lepton selection efficiency is also obtained from simulation, but corrected for any difference between the efficiency measured in data and simulation [53]. A systematic uncertainty of 50 % is assigned to the correction for contamination from background events taken from simulation.

The sources of uncertainty in the $\mathrm {W}$ $+$ jets estimation are: (1) the statistical uncertainty in the number of single-muon events in the data, (2) uncertainty in the background events obtained from simulation, (3) uncertainty in acceptance from PDFs and size of the simulation samples and uncertainty in the selection efficiency from the variation in the data/MC scale factor and size of the simulation samples. A summary of the fractional contributions of these uncertainties to the total uncertainty in the $\mathrm {W}$ $+$ jets background is shown in Table 2.

**Table 2:** Summary of the statistical and systematic contributions to the total uncertainty on the W $+$ jets background from the various factors used in the estimation from data

$E_{\mathrm {T}}^{\text {miss}}$ ($\text {GeV}$) $\rightarrow$	$>$250	$>$300	$>$350	$>$400	$>$450	$>$500	$>$550
(1) $\mathrm {W}(\mu \nu )\,$ $+$ jets statistical unc.	0.8	1.3	1.9	2.8	3.9	5.5	7.3
(2) Background	2.3	2.3	2.2	2.3	2.4	2.6	2.8
(3) Acceptance and efficiency	4.5	4.6	4.9	5.2	5.7	6.4	7.6
Total uncertainty (%)	5.1	5.3	5.7	6.4	7.3	8.8	11

The QCD multijet background is estimated by correcting the prediction from simulation with a data/MC scale factor derived from a QCD-enriched region in data. The QCD-enriched region is selected by applying the signal selection but relaxing the requirement on the jet multiplicity and the angular separation between the first and second jet and instead requiring that the azimuth angle between the $E_{\mathrm {T}}^{\text {miss}}$ and the second jet is less than 0.3. The $p_{\mathrm {T}}$ threshold for selecting jets (all except the leading jet) is varied from 20 to 80$\,\text {GeV}$ and an average scale factor is derived from a comparison between data and simulation. The ${{t{\bar{t}}}}$ background is determined from simulation and normalised to the approximate next-to-next-to-leading-order cross section [54], and is validated using a control sample of $\mathrm {e}\mu$ events in data. The predictions for the number of diboson ($\mathrm {W}\mathrm {W}$, $\mathrm {W}\mathrm {Z}$, $\mathrm {Z}\mathrm {Z}$) events are also determined from simulation, and normalised to their next-to-leading-order (NLO) cross sections [55]. Predictions for $\mathrm {W}\gamma$ and $\mathrm {Z}(\nu \nu )\gamma$ events are included in the estimation of $\mathrm {W}$ $+$ jets and $\mathrm {Z}(\nu \nu )$ $+$ jets from data, as photons are not explicitly vetoed in the estimation of the $\mathrm {W}$ $+$ jets and $\mathrm {Z}(\nu \nu )$ $+$ jets backgrounds. Single top and $\mathrm {Z}(\ell \ell )$ $+$ jets (including $\mathrm {Z}(\ell \ell )\gamma$ production) are predicted to contribute $\sim$0.3 % of the total background, and are determined from simulation. A $50~\%$ uncertainty is assigned to these backgrounds. In addition to this 50 % uncertainty, the uncertainty on the QCD background also receives a contribution of 30 % arising from the uncertainty on the data/MC scale factor.

## Results

**Fig. 4:**
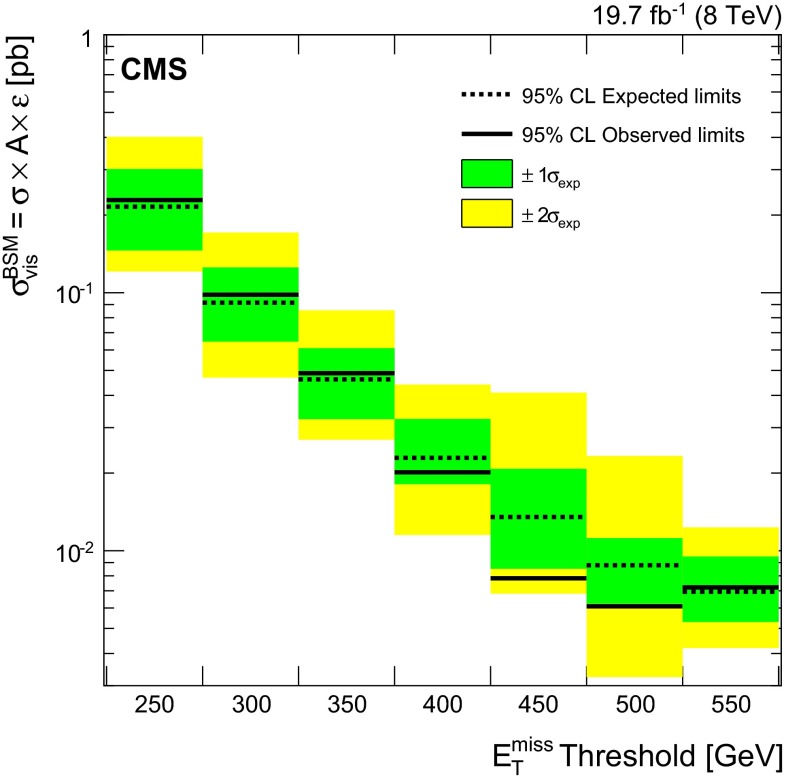
The model-independent observed and expected 95 % CL upper limits on the visible cross section times acceptance times efficiency ($\sigma \times A \times \varepsilon$) for non-SM production of events. *Shaded areas* show the $\pm 1\sigma$ and $\pm 2\sigma$ bands on the expected limits

A summary of the predictions and corresponding uncertainties for all the SM backgrounds and the data is shown in Table 3 for different values of the $E_{\mathrm {T}}^{\text {miss}}$ selection. The observed number of events is consistent with the background expectation, given the statistical and systematic uncertainties. The CL$_\mathrm {s}$ method [56–58] is employed for calculating the upper limits on the signal cross section using a profile likelihood ratio as the test-statistic and systematic uncertainties modeled by log-normal distributions. Uncertainties in the signal acceptance (described below) are taken into account when upper limits on the cross section are determined. The expected and observed 95 % confidence level (CL) upper limits on the contribution of events from new physics are also shown. The model-independent upper limits on the visible cross section for non-SM production of events (denoted $\sigma _\text {vis}^\mathrm {BSM}$) are shown in Fig. 4.

**Table 3:** SM background predictions for the numbers of events passing the selection requirements, for various $E_{\mathrm {T}}^{\text {miss}}$ thresholds, compared with the observed numbers of events. The uncertainties include both statistical and systematic components. The last two rows give the expected and observed upper limits, at 95 % CL, for the contribution of events from non-SM sources passing the selection requirements

$E_{\mathrm {T}}^{\text {miss}}$ ($\text {GeV}$ ) $\rightarrow$	$>$250	$>$300	$>$350	$>$400	$>$450	$>$500	$>$550
$\mathrm {Z}(\nu \nu )$ $+$ jets	32100 $\pm$ 1600	12700 $\pm$ 720	5450 $\pm$ 360	2740 $\pm$ 220	1460 $\pm$ 140	747 $\pm$ 96	362 $\pm$ 64
$\mathrm {W}$ $+$ jets	17600 $\pm$ 900	6060 $\pm$ 320	2380 $\pm$ 130	1030 $\pm$ 65	501 $\pm$ 36	249 $\pm$ 22	123 $\pm$ 13
$({{t{\bar{t}}}})$	446 $\pm$ 220	167 $\pm$ 84	69 $\pm$ 35	31 $\pm$ 16	15 $\pm$ 7.7	6.6 $\pm$ 3.3	2.8 $\pm$ 1.4
$\mathrm {Z}(\ell \ell )$ $+$ jets	139 $\pm$ 70	44 $\pm$ 22	18 $\pm$ 9.0	8.9 $\pm$ 4.4	5.2 $\pm$ 2.6	2.3 $\pm$ 1.2	1.0 $\pm$ 0.5
Single t	155 $\pm$ 77	53 $\pm$ 26	18 $\pm$ 9.1	6.1 $\pm$ 3.1	0.9 $\pm$ 0.4	–	–
QCD multijets	443 $\pm$ 270	94 $\pm$ 57	29 $\pm$ 18	4.9 $\pm$ 3.0	2.0 $\pm$ 1.2	1.0 $\pm$ 0.6	0.5 $\pm$ 0.3
Diboson	980 $\pm$ 490	440 $\pm$ 220	220 $\pm$ 110	118 $\pm$ 59	65 $\pm$ 33	36 $\pm$ 18	20 $\pm$ 10
Total SM	51800 $\pm$ 2000	19600 $\pm$ 830	8190 $\pm$ 400	3930 $\pm$ 230	2050 $\pm$ 150	1040 $\pm$ 100	509 $\pm$ 66
Data	52200	19800	8320	3830	1830	934	519
Exp. upper limit ${+}1\sigma$	5940	2470	1200	639	410	221	187
Exp. upper limit ${-}1\sigma$	2870	1270	638	357	168	123	104
Exp. upper limit	4250	1800	910	452	266	173	137
Obs. upper limit	4510	1940	961	397	154	120	142

The total systematic uncertainty in the signal yield is found to be approximately 20 % for the vector and axial-vector dark matter models, ADD extra dimensions, and unparticles, and between 20 and 35 % for the scalar dark matter model. The sources of systematic uncertainties considered are: jet energy scale, which is estimated by shifting the four-vectors of the jets by an $\eta$- and $p_{\mathrm {T}}$-dependent factor [32]; PDFs, evaluated using the PDF4LHC prescription from the envelope of the CT10 [59], MSTW2008NLO [60], NNPDF2.1 [61] error sets; renormalization/factorization scales, evaluated by varying simultaneously the renormalization/factorization scale up and down by a factor of 2; modeling of the ISR; simulation of event pileup; and the integrated luminosity measurement. The PDF uncertainty is also evaluated using the LO PDFs (MSTW2008LO [60] and NNPDF21LO [61]) and found to be consistent with the results from the NLO PDFs. The ISR uncertainty is estimated by varying parton shower parameters within pythia for all signal models. In addition, for the dark matter models, a further uncertainty in ISR is obtained by considering the difference in acceptance and cross section from the nominal generated samples to those where a $p_{\mathrm {T}}$ threshold of 15$\,\text {GeV}$ is applied on the generated partons and the MLM matching prescription is used to match the matrix element calculation to the parton shower in pythia, with the matching $p_{\mathrm {T}}$ scale of 20$\,\text {GeV}$. The dominant uncertainties are from the modeling of the ISR, which contributes at the level of 5 % for the dark matter models and 12 % for ADD/unparticle models, and the choice of renormalization/factorization scale, which leads to an uncertainty of around 10 % for ADD/unparticle models and 15 % for the dark matter models. In addition, the uncertainty on the scalar dark matter model is dominated by the PDF uncertainty, which ranges from 7 % for low DM mass and up to 30 % for high DM mass.

For each signal point, limits are derived from the signal region expected to give the best limit on the cross section. For dark matter and ADD models, the most stringent limits are obtained for $$E_{\mathrm {T}}^{\text {miss}}> 500$$$$\,\text {GeV}$$, whereas for unparticles the optimal selection varies from $$E_{\mathrm {T}}^{\text {miss}}> 300$$$$\,\text {GeV}$$ for $${{\Lambda _\mathrm{U}}} = 1$$$$\,{\mathrm{TeV}}\,$$to $$E_{\mathrm {T}}^{\text {miss}}> 500$$$$\,\text {GeV}$$ for larger values of $${{\Lambda _\mathrm{U}}} $$.

## Interpretation

The observed limit on the cross section depends on the mass of the dark matter particle and the nature of its interaction with the SM particles. The limits on the effective contact interaction scale $\Lambda$ as a function of $M_{\chi }$ can be translated into a limit on the dark matter-nucleon scattering cross section using the reduced mass of the $\chi$-nucleon system [9].

**Fig. 5:**
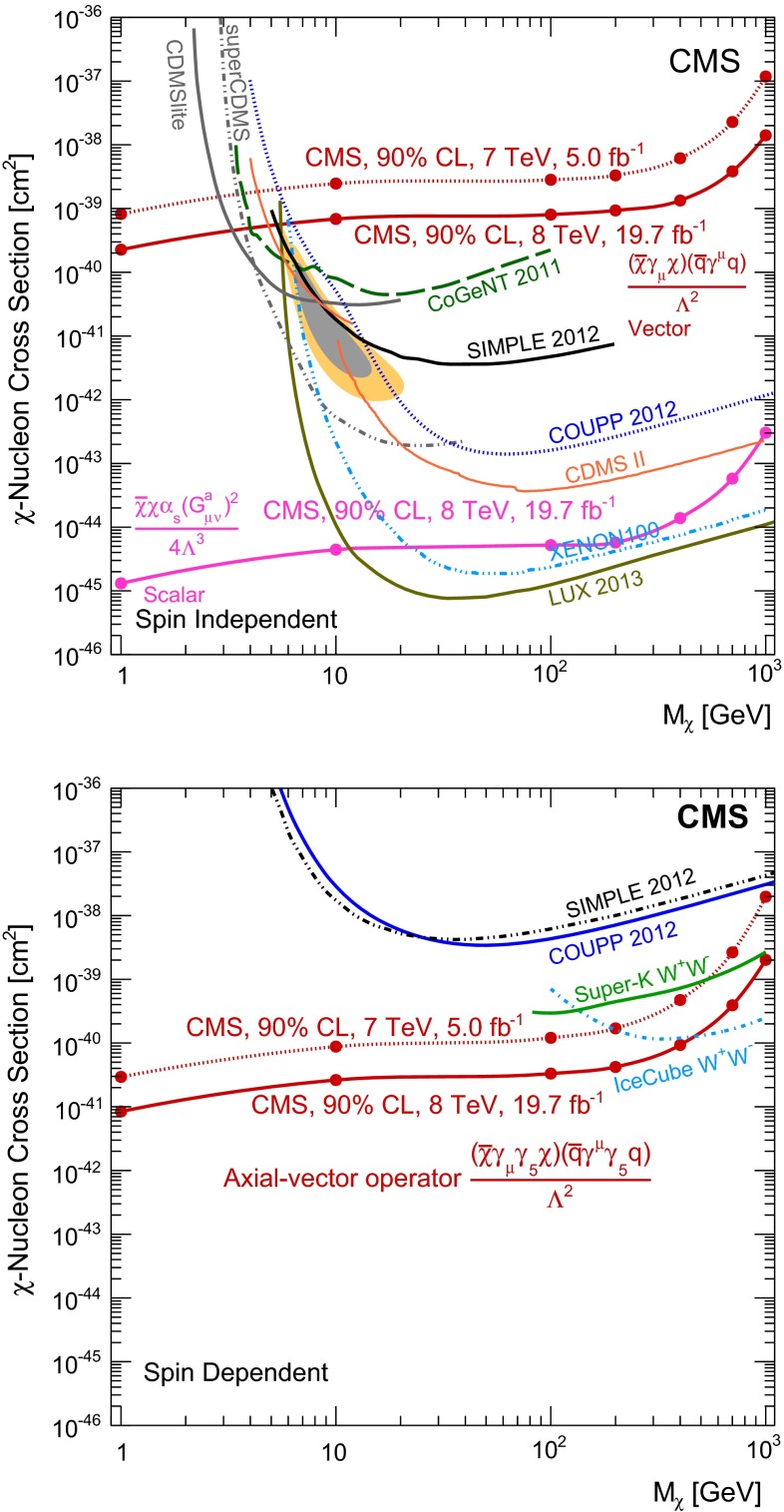
Upper limits on the DM-nucleon cross section, at 90 % CL, plotted against DM particle mass and compared with previously published results. $Top \,$limits for the vector and scalar operators from the previous CMS analysis [11], together with results from the CoGeNT [66], SIMPLE [67], COUPP [68], CDMS [69, 70], SuperCDMS [71], XENON100 [72], and LUX [73] collaborations. The *solid* and *hatched yellow contours* show the 68 and 90 % CL contours respectively for a possible signal from CDMS [74]. $Bottom \,$limits for the axial-vector operator from the previous CMS analysis [11], together with results from the SIMPLE [67], COUPP [68], Super-K [75], and IceCube [76] collaborations

Within the framework of the effective field theory, we extract limits on the contact interaction scale, $\Lambda$, and on the DM-nucleon scattering cross-section, $\sigma _{\chi \mathrm {N}}$. The confidence level chosen for these limits is 90 %, to enable a direct comparison with the results from the direct detection experiments. The expected and observed limits as a function of the DM mass, $M_{\chi }$, are shown for the vector and axial-vector operators [6, 9] in Tables 4 and 5, respectively, and for the scalar operator [6, 9] in Table 6. Figure 5 shows the 90 % CL upper limits on the DM-nucleon scattering cross section as a function of $M_{\chi }$ together with those from the direct detection experiments and the previously published CMS result. The limits for the axial-vector operator translate to spin dependent interactions of the dark matter with nucleons, and for the vector and scalar operators they translate to spin independent dark matter-nucleon interactions.

Given the high centre-of-mass energies that are being probed by the LHC, it is important to consider the possibility that the effective theory is not always valid. The validity of the effective theory has been discussed in [7, 9, 62–65]. It is pointed out in the literature that for theories to be perturbative the product of the couplings $g_{\chi }g_{\mathrm {q}}$ is typically required to be smaller than 4$\pi$, and this condition is likely not satisfied for the entire region of phase space probed by the collider searches. In addition, the range of values for the couplings being probed within the effective field theory may be unrealistically large [65].

**Fig. 6:**
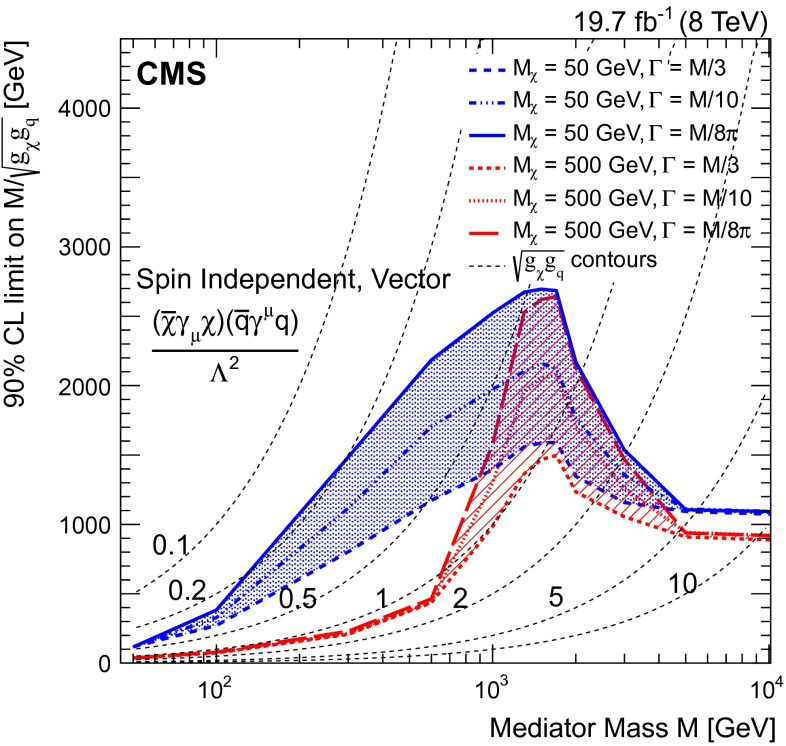
Observed limits on the mediator mass divided by coupling, $M/\!\sqrt{g_{\chi }g_{\mathrm {q}}}$, as a function of the mass of the mediator, $M$, assuming vector interactions and a *dark matter* mass of 50$\,\text {GeV}$ (*blue*, *filled*) and 500$\,\text {GeV}$ (*red*, *hatched*). The *width*, $\Gamma$, of the mediator is varied between $M/3$ and $M/8\pi$. The *dashed lines* show contours of constant coupling $\sqrt{g_{\chi }g_{\mathrm {q}}}$

Therefore, we also consider the explicit case of an $s$-channel mediator with vector interactions, following the model described in [62]. The mass of the mediator is varied for two fixed values of the mass of the DM particle, 50 and 500$\,\text {GeV}$. The width of the mediator is varied between the extremes of $M$/$8\pi$ and $M/3$, where $M/8\pi$ corresponds to a mediator that can annihilate into only one quark flavor and helicity, has couplings $g_{\chi }g_{\mathrm {q}} = 1$ and is regarded as a lower limit on the mediator width. However, not all widths may be physically realizable for the DM couplings that are considered [62]. Figure 6 shows the resulting observed limits on the mediator mass divided by coupling ($M/\!\sqrt{g_{\chi }g_{\mathrm {q}}}$), as a function of the mass of the mediator. The resonant enhancement in the production cross section, once the mass of the mediator is within the kinematic range and can be produced on-shell, can be clearly seen. The limits on $M/\!\sqrt{g_{\chi }g_{\mathrm {q}}}$ approximate to those obtained from the effective field theory framework at large mediator mass, but are weaker at low mediator mass. Also shown are dashed contours corresponding to constant values of the couplings $g_{\chi }g_{\mathrm {q}}$.

**Table 4:** Expected and observed 90 % CL upper limits on the DM-nucleon cross section, $\sigma _{\chi \mathrm {N}}$, and 90 % CL lower limits on the effective contact interaction scale, $\Lambda$, for the vector operator

$M_{\chi }$ ($\,\text {GeV}$)	Expected		Expected ${-}1\sigma$		Expected ${+}1\sigma$		Observed	
	$\Lambda$ ($\text {GeV}$)	$\sigma _{\chi \mathrm {N}}$ (cm$^{2}$)	$\Lambda$ ($\text {GeV}$)	$\sigma _{\chi \mathrm {N}}$ (cm$^{2}$)	$\Lambda$ ($\text {GeV}$)	$\sigma _{\chi \mathrm {N}}$ (cm$^{2}$)	$\Lambda$ ($\text {GeV}$)	$\sigma _{\chi \mathrm {N}}$ (cm$^{2}$)
1	951	$3.19 \times 10^{-40}$	1040	$2.23 \times 10^{-40}$	843	$5.17 \times 10^{-40}$	1029	$2.33 \times 10^{-40}$
10	959	$9.68 \times 10^{-40}$	1049	$6.77 \times 10^{-40}$	850	$1.57 \times 10^{-39}$	1038	$7.06 \times 10^{-40}$
100	960	$1.13 \times 10^{-39}$	1050	$7.92 \times 10^{-40}$	851	$1.83 \times 10^{-39}$	1039	$8.26 \times 10^{-40}$
200	926	$1.32 \times 10^{-39}$	1013	$9.21 \times 10^{-40}$	821	$2.13 \times 10^{-39}$	1003	$9.60 \times 10^{-40}$
400	848	$1.89 \times 10^{-39}$	927	$1.32 \times 10^{-39}$	752	$3.06 \times 10^{-39}$	918	$1.37 \times 10^{-39}$
700	652	$5.40 \times 10^{-39}$	713	$3.78 \times 10^{-39}$	578	$8.75 \times 10^{-39}$	706	$3.94 \times 10^{-39}$
1000	471	$1.99 \times 10^{-38}$	515	$1.39 \times 10^{-38}$	418	$3.22 \times 10^{-38}$	510	$1.45 \times 10^{-38}$

**Table 5:** Expected and observed 90 % CL upper limits on the DM-nucleon cross section, $\sigma _{\chi \mathrm {N}}$, and 90 % CL lower limits on the effective contact interaction scale, $\Lambda$, for the axial-vector operator

$M_{\chi }$ ($\text {GeV}$ )	Expected		Expected ${-}1\sigma$		Expected ${+}1\sigma$		Observed	
	$\Lambda$ ($\text {GeV}$ )	$\sigma _{\chi \mathrm {N}}$ (cm$^{2}$)	$\Lambda$ ($\text {GeV}$ )	$\sigma _{\chi \mathrm {N}}$ (cm$^{2}$)	$\Lambda$ ($\text {GeV}$ )	$\sigma _{\chi \mathrm {N}}$ (cm$^{2}$)	$\Lambda$ ($\text {GeV}$ )	$\sigma _{\chi \mathrm {N}}$ (cm$^{2}$)
1	947	$1.19 \times 10^{-41}$	1035	$8.33 \times 10^{-42}$	839	$1.93 \times 10^{-41}$	1025	$8.68 \times 10^{-42}$
10	949	$3.71 \times 10^{-41}$	1038	$2.59 \times 10^{-41}$	841	$6.00 \times 10^{-41}$	1027	$2.70 \times 10^{-41}$
100	932	$4.68 \times 10^{-41}$	1019	$3.28 \times 10^{-41}$	826	$7.58 \times 10^{-41}$	1008	$3.41 \times 10^{-41}$
200	880	$5.94 \times 10^{-41}$	962	$4.15 \times 10^{-41}$	780	$9.62 \times 10^{-41}$	952	$4.33 \times 10^{-41}$
400	722	$1.32 \times 10^{-40}$	789	$9.21 \times 10^{-41}$	640	$2.13 \times 10^{-40}$	781	$9.60 \times 10^{-41}$
700	505	$5.52 \times 10^{-40}$	552	$3.86 \times 10^{-40}$	447	$8.94 \times 10^{-40}$	546	$4.03 \times 10^{-40}$
1000	335	$2.85 \times 10^{-39}$	366	$1.99 \times 10^{-39}$	297	$4.61 \times 10^{-39}$	363	$2.08 \times 10^{-39}$

**Table 6:** Expected and observed 90 % CL upper limits on the DM-nucleon cross section, $\sigma _{\chi \mathrm {N}}$, and 90 % CL lower limits on the effective contact interaction scale, $\Lambda$, for the scalar operator

$M_{\chi }$ ($\text {GeV}$ )	Expected		Expected ${-}1\sigma$		Expected ${+}1\sigma$		Observed	
	$\Lambda$ ($\text {GeV}$)	$\sigma _{\chi \mathrm {N}}$ (cm$^{2}$)	$\Lambda$ ($\text {GeV}$ )	$\sigma _{\chi \mathrm {N}}$ (cm$^{2}$)	$\Lambda$ ($\text {GeV}$ )	$\sigma _{\chi \mathrm {N}}$ (cm$^{2}$)	$\Lambda$ ($\text {GeV}$ )	$\sigma _{\chi \mathrm {N}}$ (cm$^{2}$)
1	411	$1.85\times 10^{-45}$	437	$1.30\times 10^{-45}$	380	$3.00\times 10^{-45}$	436	$1.31\times 10^{-45}$
10	407	$6.15\times 10^{-45}$	432	$4.31\times 10^{-45}$	375	$1.02\times 10^{-44}$	430	$4.44\times 10^{-45}$
100	407	$7.25\times 10^{-45}$	432	$5.08\times 10^{-45}$	375	$1.20\times 10^{-44}$	430	$5.23\times 10^{-45}$
200	402	$7.96\times 10^{-45}$	426	$5.58\times 10^{-45}$	369	$1.31\times 10^{-44}$	424	$5.75\times 10^{-45}$
400	348	$1.90\times 10^{-44}$	368	$1.34\times 10^{-44}$	319	$3.16\times 10^{-44}$	366	$1.39\times 10^{-44}$
700	274	$7.91\times 10^{-44}$	290	$5.60\times 10^{-44}$	252	$1.32\times 10^{-43}$	289	$5.79\times 10^{-44}$
1000	208	$4.15\times 10^{-43}$	220	$2.94\times 10^{-43}$	191	$6.93\times 10^{-43}$	219	$3.04\times 10^{-43}$

**Fig. 7:**
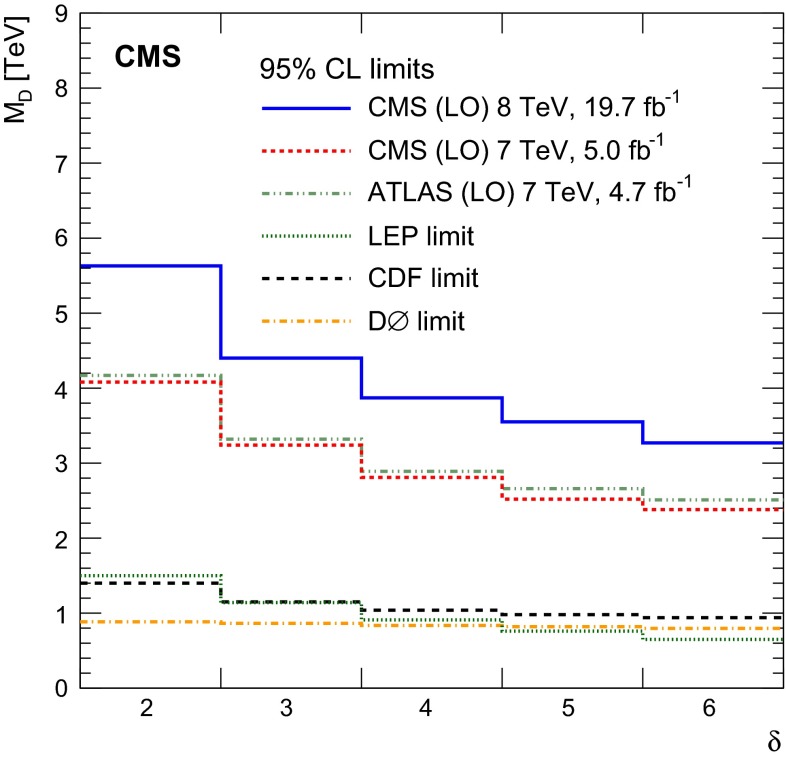
Lower limits at 95 % CL on ${M_\mathrm {D}}$ plotted against the number of extra dimensions $\delta$, with results from the ATLAS [25], CMS [11], LEP [19–21, 78], CDF [22], and DØ [23] collaborations

Lower limits on ${M_\mathrm {D}}$ in the ADD model, for different values of $\delta$, have been obtained using LO cross section calculations, and the application of NLO QCD corrections, using $K$-factors, $K = \sigma _\mathrm {NLO}/\sigma _\mathrm {LO}$ of 1.4 for $\delta = \{2, 3\},$ 1.3 for $\delta = \{4,$ 5}, and 1.2 for $\delta = 6$ [77]. Figure 7 shows 95 % CL limits at LO, compared to published results from ATLAS, LEP, and the Tevatron. The ATLAS limits were produced using the full kinematic phase space, without any truncation applied to restrict the phase space to the region where the effective field theory is valid. The CMS limits are obtained using the truncated phase space, after discarding events for which the parton center of mass energy $\hat{s} > {M_\mathrm {D}}^2$. The maximum difference in the cross section evaluated with and without the truncation was found to be 11 %. Table 7 shows the expected and observed limits at LO and NLO for the ADD model.

**Table 7:** Expected and observed 95 % CL lower limits on ADD model parameter $M_\mathrm {D}$ in $\text {TeV}$ as a function of $\delta$ at LO and NLO

$\delta$	Expected limit	${+}1\sigma$	${-}1\sigma$	Observed limit
LO limit on $M_\mathrm {D}$ ($\text {TeV}$)				
2	5.09	4.80	5.60	5.61
3	3.99	3.87	4.36	4.38
4	3.74	3.56	3.86	3.86
5	3.32	2.99	3.54	3.55
6	2.99	2.98	3.25	3.26
NLO limit on ${M_\mathrm {D}}$ ($\text {TeV}$)				
2	5.53	5.21	6.08	6.09
3	4.34	4.21	4.74	4.77
4	3.85	3.66	3.97	3.97
5	3.49	3.14	3.72	3.73
6	3.24	3.23	3.52	3.53

**Fig. 8:**
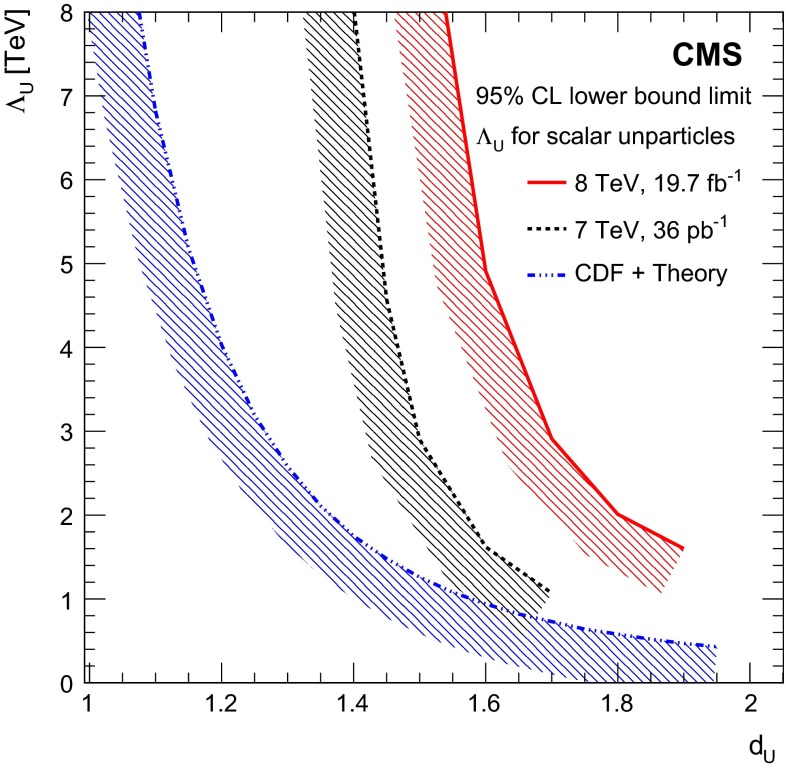
The expected and observed lower limits on the unparticle model parameters ${{\Lambda _\mathrm{U}}}$ as a function of $d_\mathrm {U}$ at 95 % CL, compared to previous results [24, 79]. The *shaded region* indicates the *side of the curve* that is excluded

Figure 8 shows the expected and observed 95 % CL limits on the cross-sections for scalar unparticles ($\mathrm {S} = 0$) with $d_\mathrm {U} = 1.5,$ 1.6, 1.7, 1.8, and 1.9 as a function of ${{\Lambda _\mathrm{U}}}$ for a fixed coupling constant $\lambda = 1$. The observed 95 % CL limit ${{\Lambda _\mathrm{U}}}$ for these values of $d_\mathrm {U}$ is shown in Table 8.

**Table 8:** Expected and observed 95 % CL lower limits on ${{\Lambda _\mathrm{U}}}$ (in $\text {TeV}$) for scalar unparticles with $d_\mathrm {U} =$1.5, 1.6, 1.7, 1.8 and 1.9 and a fixed coupling constant $\lambda = 1$

$d_\mathrm {U}$	Expected limit on ${{\Lambda _\mathrm{U}}}$ (TeV)	${+}1\sigma$	${-}1\sigma$	Observed limit on ${{\Lambda _\mathrm{U}}}$ ($\text {TeV}$)
1.5	7.88	6.63	8.39	10.00
1.6	3.89	2.51	4.88	4.91
1.7	2.63	2.09	2.89	2.91
1.8	1.91	1.76	1.98	2.01
1.9	1.41	0.88	1.46	1.60

## Summary

A search for particle dark matter, large extra dimensions, and unparticle production has been performed in the monojet channel using a data sample of proton–proton collisions at $$\sqrt{s} = 8$$$$\,{\mathrm{TeV}}\,$$corresponding to an integrated luminosity of 19.7$$\,\text {fb}^\text {-1}$$. The dominant backgrounds to this topology are from $$\mathrm {Z}(\nu \nu )$$ $$+$$ jets and $$\mathrm {W}(\ell \nu )\, +$$ jets events, and are estimated from data samples of $$\mathrm {Z}(\mu \mu )$$ and $$\mathrm {W}(\mu \nu )\,$$events, respectively. The data are found to be in agreement with expected contributions from standard model processes. Limits are set on the DM-nucleon scattering cross section assuming vector, axial-vector, and scalar operators. Limits are also set on the fundamental Planck scale $${M_\mathrm {D}}$$ in the ADD model of large extra dimensions and on the unparticle model parameter $${{\Lambda _\mathrm{U}}} $$. Compared to previous CMS publications in this channel, the lower limits on $${M_\mathrm {D}}$$ represent an approximately 40 % improvement, and the lower limits on the unparticle model parameter $${{\Lambda _\mathrm{U}}} $$ represent an improvement by a factor of roughly 3. The upper limit on the DM-nucleon cross section has been reduced from $$8.79\times 10^{-41}$$ to $$2.70\times 10^{-41}\,\text {cm} ^{2}$$ for the axial-vector operator and from $$2.47\times 10^{-39}$$ to $$7.06\times 10^{-40}\,\text {cm} ^{2}$$ for the vector operator for a particle DM mass of 10$$\,\text {GeV}$$. The constraints on ADD models and unparticles are the most stringent limits in this channel and those on the DM-nucleon scattering cross section are an improvement over previous collider results.
